# Characterization of a novel LQT3 variant with a selective efficacy of mexiletine treatment

**DOI:** 10.1038/s41598-019-49450-0

**Published:** 2019-09-10

**Authors:** Hyun-Ji Kim, Bok-Geon Kim, Jong Eun Park, Chang-Seok Ki, June Huh, Jae Boum Youm, Jong-Sun Kang, Hana Cho

**Affiliations:** 1 Department of Physiology, Sungkyunkwan University, Suwon, Korea; 2 Department of Molecular Cell Biology, Sungkyunkwan University, Suwon, Korea; 3 Single Cell Network Research Center, Sungkyunkwan University, Suwon, Korea; 4 Department of Laboratory Medicine, Hanyang University Guri Hospital, Hanynag University College of Medicine, Guri, Korea; 5 GC Genome, Yongin, Korea; 6 Division of Pediatric Cardiology, Department of Pediatrics, Samsung Medical Center, Sungkyunkwan University School of Medicine, Seoul, Korea; 7 Department of Physiology, College of Medicine, Cardiovascular and Metabolic Disease Center, Inje University, Busan, Korea

**Keywords:** Physiology, Ventricular tachycardia

## Abstract

Pathogenic variants in the human *SCN5A* gene encoding the *a*-subunit of the principle Na^+^ channel (Nav1.5) are associated with long QT syndrome (LQTS) 3. LQT3 patients display variable responses to Na^+^ channel blockers demanding for the development of variant-specific therapeutic strategies. Here we performed a combined electrophysiological analysis with in silico simulation of variant channel to elucidate mechanisms of therapeutic responsiveness. We identified a novel *SCN5A* variant (A1656D) in a LQTS patient with a distinct response to mexiletine resulting in suppression of non-sustained ventricular tachycardia and manifestation of premature atrial contraction. Patch clamp analysis revealed that A1656D variant exerted gain-of-function effects including hyperpolarizing shift of the voltage-dependence of activation, depolarizing shift in the voltage-dependence of inactivation, and slowing of fast inactivation. Among ranolazine, flecainide, and mexiletine, only mexiletine restored inactivation kinetics of A1656D currents. In silico simulation to assess the effect of A1656D variant on ventricular cardiac cell excitation predicted a prolonged action potential which is consistent with the prolonged QT and non-sustained ventricular tachycardia of the patient. It also predicted that only mexiletine suppressed the prolonged action potential of human ventricular myocytes expressing A1656D. These data elucidate the underlying mechanism of the distinct response to mexiletine in this patient.

## Introduction

The voltage-gated Na^+^ channel is an integral membrane protein, a part of a macromolecular complex that is central to signaling in excitable tissues such as hearts. It consists of various subunits, but only the principal (α) subunit is required for the channel function. Ten different mammalian α subunits (*SCN1A-SCN11A*) have been cloned and characterized. The Na^+^ channelopathies were among the first molecularly characterized human ion channel diseases^1^. Mutations in Nav1.5 channel subunits underlie the inherited arrhythmias including long QT syndrome (LQTS), Brugada syndrome, cardiac conduction disease, sudden infant death syndrome and cardiomyopathy^2,3^. LQTS is a relatively uncommon (1 in 2500) genetic disorder associated with life-threatening arrhythmias that has provided a wealth of information about fundamental mechanisms underlying human cardiac electrophysiology^4^. LQTS is currently associated with mutations in more than 15 different genes^5–7^. LQT variant 3 (LQT3) is caused by mutations in SCN5A and accounts for approximately 10% of LQTS patients^8^.

LQT3 is characterized by a severe prognosis^8,9^ and an incomplete response to beta-blockers^10^, as compared to LQTS type 1, the most common LQTS variant. Since the arrhythmic events in LQT3 occur predominantly at rest^11^, the efficacy of beta-blocker therapy is uncertain^10,12^. In addition, multiple cases have reported mixed efficacy of Na^+^ channel blockers applied to LQT3 patients^13–15^, which complicates to select the proper therapeutics. Thus pharmacological targeting of mutation-altered Na^+^ channels appears to be promising therapeutic strategy to manage this LQT3 variant. Personalized approach to manage arrhythmia is a well-established concept, given that each patient harbors a unique mutation that influences the risk, onset, and progression of their disease. For any specific mutation of Na^+^ channel, the responses of mutation carrier to a given treatment regimen might differ, largely driven by the unique Na^+^ channel phenotype caused by different mutations. Therefore, developing a personalized therapy strategy to ensure an optimal outcome for individual patients requires the mechanistic understanding of distinct efficacy of antiarrhythmic drug for the patients at the molecular and cellular levels. Characterization of clinical phenotypes and therapeutic responses for Na^+^ channel harboring specific mutations is actively pursued; however, many of the relevant studies have been limited by the fact that they were performed in expression systems rather than native cells.

To circumvent this limitation, the combined approach of experimental analysis with in silico simulation using human atrial and ventricular myocytes model is beginning to emerge^16,17^. In this study, we identified a de novo SCN5A mutation in a newborn with QT prolongation and non-sustained ventricular tachycardia (NSVT). *In vitro* expression study and in silico simulation revealed that the gain-of-function effect of A1656D mutation is consistent with the pronounced phenotype of the patient. Furthermore, our data show that out of 3 tested antiarrhythmic drugs, mexiletine effectively recovers the channel function only in ventricular cells while it fails to suppress atrial arrhythmia. These are well correlated with the suppression of NSVT and the occurrence of premature atrial contraction (PAC) in the patient post mexiletine therapy, supporting for the importance of understanding the biophysical and pharmacological characterization of a distinct mutant to develop a mutation-specific therapeutic approach to manage arrhythmias.

## Materials and Methods

### Patients and clinical investigations

Patient was evaluated by clinical examination, 12-lead ECG, echocardiography, and 24 h Holter recording.

### Standard protocol approvals, registrations, and patient consents

We obtained written informed consent from each participant. This study was approved by the Institutional Review Board at in the Sungkyunkwan University School of Medicine (SUSM) and Samsung Medical Center. In addition, all methods were carried out in accordance with the approved guidelines.

### Genetic analysis

Genomic DNA was isolated from peripheral blood leukocytes using the Wizard Genomic DNA Purification kit (Promega, Madison, WI, USA). All exons and their flanking intronic regions of *SCN5A* were amplified by polymerase chain reaction using primers designed by the authors (available on request), and Sanger sequencing was performed using an ABI Prism 3730xl Genetic Analyzer (Applied Biosystems, Foster City, CA, USA). To assess the frequency of a variant in control population, we used the Genome Aggregation Database (gnomAD) (http://gnomad.broadinstitute.org/).

### Generation of SCN5A mutant and transfection in HEK293T cells

The SCN5A-IRES GFP plasmid was kindly provided by Dr. Tomaselli. The mutation of *SCN5A* A1656D was generated by site directed mutagenesis using the QuickChange Site-Directed Mutagenesis kit (Agilent Technologies, CA, USA) and the mutation was confirmed by sequence analysis. HEK 293T cells (ATCC, CRL-3216) were cultured in 10% FBS (fetal bovine serum) in DMEM (Dulbecco’s Modified Eagle Medium) with antibiotics. For transfection experiment, equal amount of Na^+^ channel α subunit and hβ1 by using Lipofectamine 2000 reagents (Invitrogen, CA, USA) and green fluorescent protein was used as the expression reporter.

### Electrophysiology

Current measurements were made with the whole cell patch-clamp technique. Voltage clamp was performed by using an EPC-10 amplifier (HEKA Instrument, Lambrecht/Pfalz, Germany) and filtered at 10 kHz. The patch pipettes (World Precision Instruments, Inc., FL, USA) were made by a Narishige puller (PP-830, Narishige Co, Ltd., Tokyo, Japan). The patch pipettes used had a resistance of 2–3 MΩ when filled with the pipette solutions. Membrane capacitance (C_m_) and series resistance (R_s_) were compensated after patch rupture; capacitance was typically 11–19 pF and access resistance was typically <10 MΩ. The normal external solution for HEK293T cell recording was as follows (in mM): 145 NaCl, 4 KCl, 10 HEPES, 10 glucose, 1 MgCl_2_, 1.8 CaCl_2_, pH 7.4 adjusted with NaOH. The pipette solution was as follows (in mM): 135 CsCl, 5 NaCl, 5 EGTA, 5 Mg-ATP, 10 HEPES, pH 7.2 adjusted with CsOH.

Currents were analyzed and fitted using Patch master (HEKA Instrument, Lambrecht/Pfalz, Germany) and Origin 6.1 (Originlab Corp., MA, USA) software. All values are given as mean ± SEM. Statistical analysis: Origin 6.1 software (Microcal Software, Inc., Northampton, MA, USA) was used for data analysis. The results are presented as the mean ± standard error. Paired or independent Student’s t-tests were used to test for significance where appropriate. P values < 0.05 were considered statistically significant. Current densities (pA/pF) were obtained after normalization to cell surface area calculated by Patch master. Late Na^+^ channel currents (I_NaL_) were measured during a 500 ms step pulse to −20 mV from a holding potential of −120 mV. To generate activation curves, cells were voltage-clamped at a holding potential (Vh) of −80 mV, and currents were elicited by depolarizing pulses of 50 ms from −60 mV to 10 mV in 5 mV increments after a strong hyperpolarizing prepulse (−120 mV, 500 ms) for a full availability of Na^+^ channels. Current density was calculated by normalizing to cell capacitance. Activation curves were obtained by transforming current data to conductance (G), which was calculated from the equation *G*_Na_ = *I/*(*V* − *E*_rev_), where: *I* is the peak Na^+^ current elicited by the depolarizing test potential; *V* is the test potential; and *E*_rev_ is the calculated Na^+^ reversal potential. The ratio *G/G*_max_ was plotted against the membrane potential and fitted with the Boltzmann equation of the form: *G/G*_max_ = (1 + exp[(*V* − *V*_1/2_)/*k*])^−1^, where *G*_max_ is the extrapolated maximum conductance, *V* is the test voltage, *V*_1/2_ is the half-activation voltage and *k* is the slope factor. Standard two-pulse protocols were used to generate the steady-state inactivation curves: from a holding potential of −100 mV, cells were stepped to 500-ms preconditioning potentials varying between −130 and −40 mV (prepulse) in 5 mV increments, followed by a 20-ms test pulse to −20 mV. Currents (I) were normalized to *I*_max_ and fit to a Boltzmann function of the form *I*/*I*_max_ = 1/(1 + exp((*V*_m_/*V*_1/2_)/*k*)) in which *V*_1/2_ is the voltage at which half of Nav1.5 channels are inactivated, *k* is the slope factor and *V*_m_ is the membrane potential.

### Computational model of *I*_Na_

A model of *I*_*Na*_ was developed by analyzing patch-clamp data of transfected SCN5A on HEK293T cells. In brief, The *I*_Na_ evoked by various pulse protocols were analyzed to get the steady-state activation (*m*_∞_) and inactivation (*h*_∞_) and time constants of activation (*τ*_*m*_) and inactivation (*τ*_*h*_) at membrane potentials between −60 and +50 mV. As for h-gate, it was divided into two groups with fast (*h*_*f*_) and slow (*h*_*s*_) kinetics, respectively. The curves of steady-steady activation and inactivation and their time constants are dependent on voltage and were fitted to following equations,

$$\begin{array}{c}{{\rm{m}}}_{\infty }=\sqrt[3]{1/(1+{e}^{(V-{V}_{{\rm{h}}({\rm{m}}\infty )})/{k}_{{\rm{m}}}})}\\ {{\rm{m}}}_{\tau }={a}_{{\rm{m}}}/({e}^{{b}_{{\rm{m}}}(V-{V}_{{{\rm{h}}}_{{m}_{\tau }}})/RT}+{e}^{-{c}_{{\rm{m}}}(V-{V}_{{{\rm{h}}}_{{m}_{\tau }}})/RT})+{d}_{{\rm{m}}}\\ {{\rm{h}}}_{{f}_{\infty }}={{\rm{h}}}_{{s}_{\infty }}=1/(1+{e}^{(V-{V}_{{\rm{h}}({\rm{h}}\infty )})/{k}_{{\rm{h}}}})\\ {{\rm{h}}}_{{f}_{\tau }}={a}_{{{\rm{h}}}_{f}}/({e}^{{b}_{{{\rm{h}}}_{f}}(V-{V}_{{{\rm{h}}}_{{f}_{\tau }}})/RT}+{e}^{-{c}_{{{\rm{h}}}_{f}}(V-{V}_{{{\rm{h}}}_{{f}_{\tau }}})/RT})+{d}_{{{\rm{h}}}_{f}}\\ {{\rm{h}}}_{{s}_{\tau }}={a}_{{{\rm{h}}}_{s}}/({e}^{{b}_{{{\rm{h}}}_{s}}(V-{V}_{{{\rm{h}}}_{{s}_{\tau }}})/RT}+{e}^{-{c}_{{{\rm{h}}}_{s}}(V-{V}_{{{\rm{h}}}_{{s}_{\tau }}})/RT})+{d}_{{{\rm{h}}}_{s}}\end{array}$$

where V is membrane potential (mV), *R* is gas constant (=8.31 *j mol*^−1^ *K*^−1^), and *T* is absolute temperature in degrees Kelvin. Those variables were then used to calculate forward (*α*) and backward rate constants (*β*) of gate m and h (*h*_*f*_ and *h*_*s*_) as follows.

$$\begin{array}{c}{\alpha }_{{\rm{m}}}={\rm{m}}/{\tau }_{{\rm{m}}}\\ {\beta }_{{\rm{m}}}=(1-{\rm{m}})/{\tau }_{{\rm{m}}}\\ {\alpha }_{{{\rm{h}}}_{f}}={{\rm{m}}}_{f}/{\tau }_{{{\rm{h}}}_{f}}\\ {\beta }_{{{\rm{h}}}_{f}}=(1-{{\rm{h}}}_{f})/{\tau }_{{{\rm{h}}}_{f}}\\ {\alpha }_{{{\rm{h}}}_{s}}={{\rm{m}}}_{s}/{\tau }_{{{\rm{h}}}_{s}}\\ {\beta }_{{{\rm{h}}}_{s}}=(1-{{\rm{h}}}_{s})/{\tau }_{{{\rm{h}}}_{s}}\end{array}$$

Relative contribution of two groups of h-gate to total open probability was expressed as *r*_*f*_ and *r*_*s*_ (=1 − *r*_*f*_), respectively.

Those rate constants were used to calculate m and h as time- and voltage-dependent variables.

Finally, the current amplitude (*T*_*Na*_) was calculated as follows, where *G*_*Na*_ is channel conductance

(nS), and *E*_*rev*_ is the reversal potential of *T*_*Na*_ (mV).

$${I}_{{\rm{Na}}}={G}_{{{\rm{Na}}}_{max}}\times {m}^{3}\times ({r}_{f}\times {h}_{f}+{r}_{s}\times {h}_{s})\times (V-{E}_{{\rm{rev}}})$$

Maximum *G*_Na_ (${G}_{{{\rm{Na}}}_{max}}$) of human ventricular myocytes were assumed to be 1125.0 (WT), 213.66 (A1656D), 231.18 (mexiletine), 259.03 (flecainide), and 261.05 nS (ranolazine), respectively. ${G}_{{{\rm{Na}}}_{max}}$ of human atrial myocytes were simply scaled down (6/100) from those of human ventricular myocytes. All associated constants of m- and h-gates are tabulated in Tables 1, 2, 3 and 4.

**Table 1:** Associated constants of m-gate.

	${{\boldsymbol{V}}}_{{\bf{h}}(m{\boldsymbol{\infty }})}$	*k*_m_	${{\boldsymbol{V}}}_{{{\bf{h}}}_{{{\boldsymbol{m}}}_{{\boldsymbol{\tau }}}}}$	*a*_m_	*b*_m_	*c*_m_	*d*_m_
WT	−36.445	−4.519	−52.000	1.206	−46.861	−126.579	0.226
A1656D	−44.694	−5.369	−68.764	5.078	196.176	85.086	0
Mexiletine	−47.173	−4.998	−62.604	6.202	256.571	126.690	0.106
Flecainide	−54.609	−3.615	−61.536	1.347	122.632	53.127	0.394
Ranolazine	−50.270	−4.950	−63.290	3.394	161.294	90.427	0.135

**Table 2:** Associated constants of *h*_*f*_-gate.

	${{\boldsymbol{V}}}_{{\bf{h}}(h{\boldsymbol{\infty }})}$	*k*_h_	${{\boldsymbol{V}}}_{{{\bf{h}}}_{{{\boldsymbol{f}}}_{{\boldsymbol{\tau }}}}}$	${{\boldsymbol{a}}}_{{{\bf{h}}}_{{\boldsymbol{f}}}}$	${{\boldsymbol{b}}}_{{{\bf{h}}}_{{\boldsymbol{f}}}}$	${{\boldsymbol{c}}}_{{{\bf{h}}}_{{\boldsymbol{f}}}}$	${{\boldsymbol{d}}}_{{{\bf{h}}}_{{\boldsymbol{f}}}}$
WT	−78.929	4.409	−50.000	0.980	117.620	96.289	0.314
A1656D	−69.476	4.898	−36.690	13.568	−181.416	2.772	0
Mexiletine	−71.958	5.486	−17.961	12.620	13.700	82.331	0
Flecainide	−73.533	8.220	53.838	11.021	168.053	35.413	0
Ranolazine	−70.530	4.866	14.483	19.607	30.882	84.456	1.745

**Table 3:** Associated constants of h_*s*_-gate.

	${{\boldsymbol{V}}}_{{{\bf{h}}}_{{{\boldsymbol{s}}}_{{\boldsymbol{\tau }}}}}$	${{\boldsymbol{a}}}_{{{\bf{h}}}_{{\boldsymbol{s}}}}$	${{\boldsymbol{b}}}_{{{\bf{h}}}_{{\boldsymbol{s}}}}$	${{\boldsymbol{c}}}_{{{\bf{h}}}_{{\boldsymbol{s}}}}$	${{\boldsymbol{d}}}_{{{\bf{h}}}_{{\boldsymbol{s}}}}$
WT	−3.000	57.314	104.658	59.032	0.0
A1656D	−3.423	449.708	128.855	165.944	17.551
Mexiletine	−10.432	89.477	48.360	160.984	6.627
Flecainide	−10.138	9.198	−15.989	362.571	17.084
Ranolazine	6.126	91.069	−103.071	−84.140	5.697

**Table 4:** Associated constants of *r*_*f*_ and *r*_*s*_.

	*r*_*f*_	*r*_*s*_
WT	0.990	0.010
A1656D	0.863	0.137
Mexiletine	0.902	0.098
Flecainide	0.500	0.500
Ranolazine	0.880	0.120

### Statistical analysis

Origin 6.1 software (Microcal Software, Inc., Northampton, MA, USA) was used for data analysis. The results are presented as the mean ± standard error. Paired or independent Student’s t-tests were used to test for significance where appropriate. P values < 0.05 were considered statistically significant.

## Results

### The clinical analysis and mutation screening

**Figure 1:**
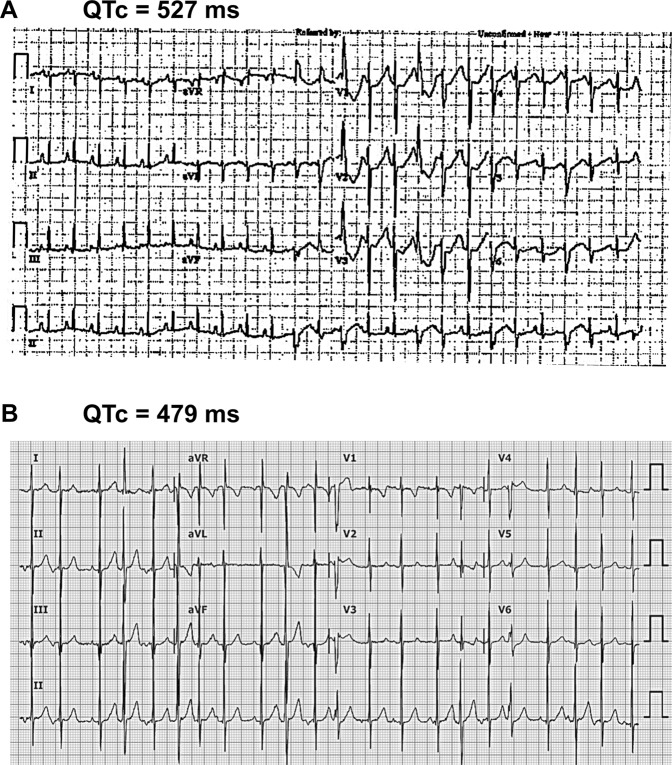
Twelve-lead electrocardiogram (ECG) from a patient. (**A**) Representative ECG from proband show a prolonged QTc interval (QTc 527 ms) and multiple ventricular premature complex at birth. (**B)** Representative ECG of the patient after the treatment with mexiletine shows improved QTc interval (QTc 479 ms) and frequent atrial premature contractions. Speed: 25 mm/sec.

A male infant was born after 37 weeks’ gestation to a 40 years old mother, who had been treated throughout pregnancy with flecainide, 100 mg bid, to treat PAC and non-sustained atrial tachycardia observed by fetal echocardiographic examination. The initial examination after birth revealed a prolonged QTc interval (approximately 527 ms) assessed by multiple electrocardiograms (ECG) (Fig. 1A). A Holter monitoring showed ventricular premature complex (VPC) and NSVT. Thus, LQT was suspected. Since the initial treatment with beta blocker and flecainide did not suppress VT, the treatment with mexiletine was started (5 mg/kg/day). Mexiletine treatment shortened the QTc interval from 527 ms to 479 ms. No further episodes of VT occurred after mexiletine treatment but PAC was still detected (Fig. 1B).

**Figure 2:**
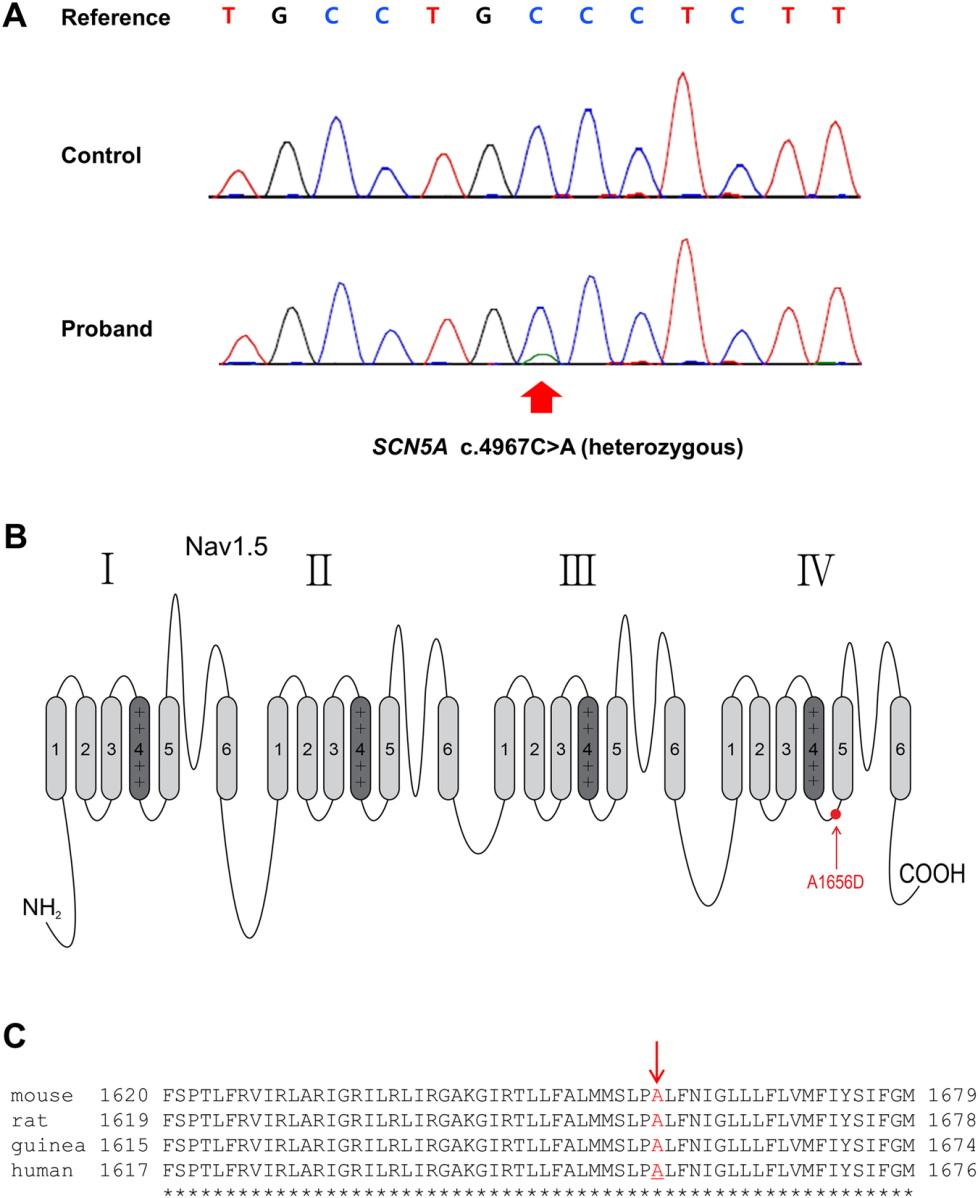
Novel A1656D mutation in the SCN5A gene. (**A**) The sequencing analysis revealed an alanine to aspartic acid mutation at 1656 site in the SCN5A gene (A1656D). **(B)** The schematic topology of Nav1.5 protein. The A1656D mutation (red circle) was located at the intracellular region between segment 4 and 5 of domain 4. **(C)** The amino acid sequence alignment showing conservation of Ala1656 (red arrow) of SCN5A in multiple species.

Based on the Sanger sequencing, we have identified a novel missense mutation in a LQT3 gene SCN5A (Fig. 2A). This mutation was caused by a transversion of C to A at position 4967 (c.4967C > A), resulting in an amino acid substitution of alanine with aspartic acid at codon 1656 of the protein (A1656D) within the intracellular region between segments 4 and 5 of domain 4 (Fig. 2B). This alanine residue is conserved among multiple species (Fig. 2C). To assess the frequency of this variant in control population, we used genetic data from 1,909 Korean individuals, as part of the Genome Aggregation Database (gnomAD) (http://gnomad.broadinstitute.org/). No single nucleotide polymorphism at this site was observed in any of control population database. Since the mutation (A1656D) was not observed in both parents, it is likely to be a de novo mutation (Table 5). In addition, the Sanger sequencing results showed that no mutations in major ion channels and transporters such as KCNQ1 (codes for the α subunit of the slowly activating IKs channel), KCNE1 (codes for the β subunit of the IKs potassium channel), KCNH2 (codes for the α subunit of the rapidly activating IKr channel) or KCNE2 (codes for the β subunit of the IKr potassium channel) or other channel-related LQTS genes were identified. Thus, we concluded that this mutation might be responsible for the observed phenotype of the patient.

**Table 5:** De novo mutation in the sodium channel gene SCN5A in our patient.

Nucleotide Change	Amino Acid Change	Control (1,909 Korean) Count (%)	Parents (2 individuals) Count (%)
c.4967C > A	p.A1656D	0	0

### A1656D is a gain of Na^+^ channel function mutation

**Figure 3:**
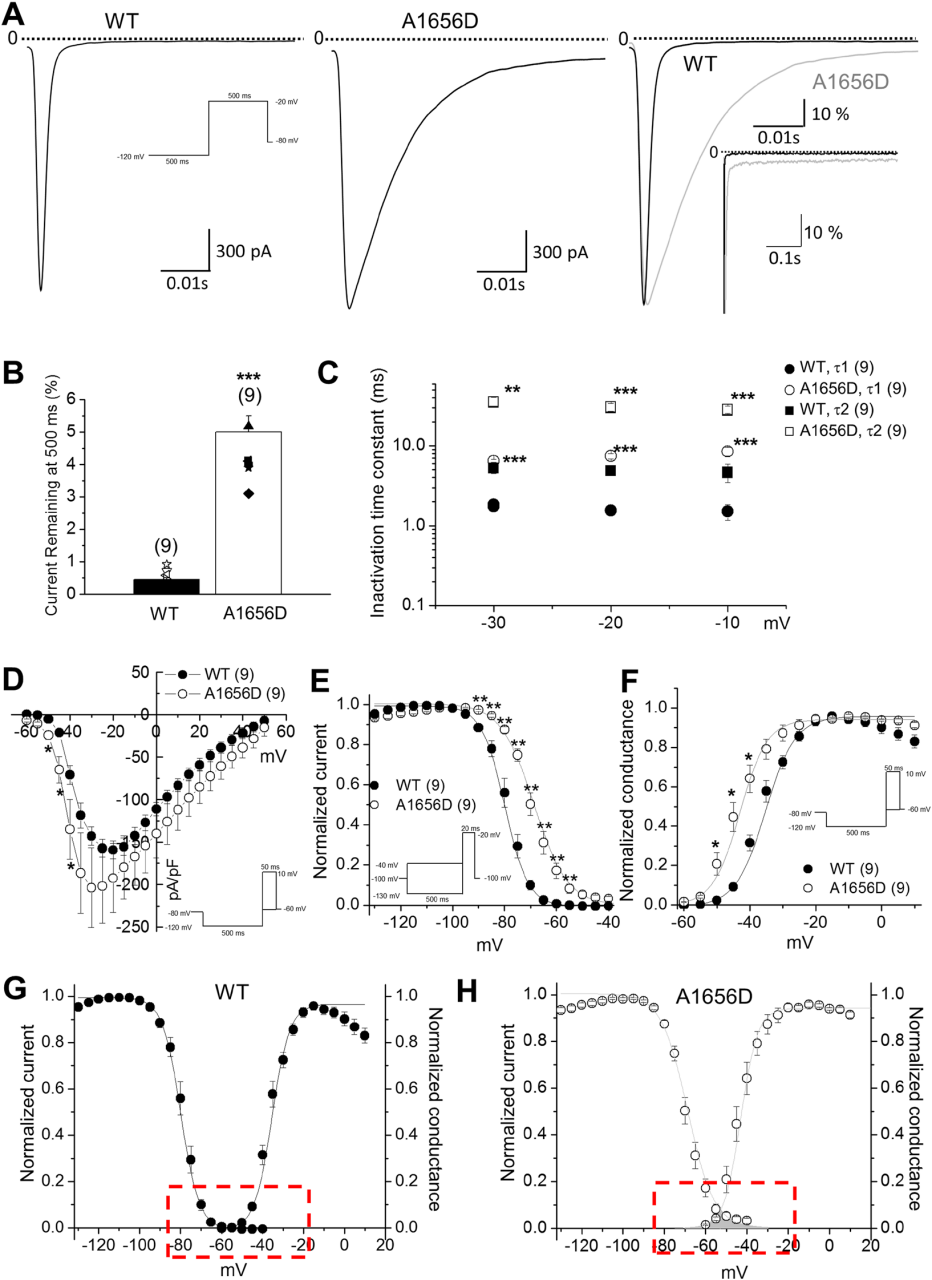
A1656D mutant channel exhibits defective I_Na_ inactivation consistent with a LQT3 phenotype. (**A**) Whole-cell Na^+^ channel currents from HEK293T cells expressing WT (left) or A1656D Nav1.5 (middle) with the β1 subunits at −20 mV (500 ms pulses applied from −120 mV holding potential at 0.2 Hz). WT and A1656D currents normalized to peak current and superimposed at high (right) and low (inset) gain. (**B)** Bar graph representing mean ± S.E.M. ***P < 0.001, Student’s t-test. percentage of current remaining at 500 ms with respect to peak current for both WT and A1656D Nav1.5. (**C)** Data represent mean ± S.E.M. **P < 0.01, ***P < 0.001, Student’s t-test. The fast and slow time constants (Tau1, Tau2) for inactivation are plotted for WT and A1656D Na^+^ channel. (**D)** Data represent mean ± S.E.M. *P < 0.05, Student’s t-test. The current-voltage (I-V) relationship for WT and A1656D currents. (**E)** Data represent mean ± S.E.M. **P < 0.01, ***P < 0.001, Student’s t-test. Steady-state inactivation was measured with a 500 ms conditioning pulse followed by a brief −20 mV test pulse. **F**. Data represent mean ± S.E.M. *P < 0.05, Student’s t-test. Steady-state activation curves, with relative conductance derived from maximal chord conductance and reversal potential (E_rev_) for each I–V, and peak I_Na_/(E_m_ − E_rev_). The resulting conductance was normalized to the maximal chord conductance. (**G–H)** Window currents of WT (**G**) and A1656D (**H**) channels. Data represent mean ± S.E.M.

To investigate the effect of A1656D on the channel activity, we expressed the wild type (WT) or A1656D mutant with WT β1 subunit in HEK293T cells and recorded whole cell Na^+^ currents by patch clamp technique. When Na^+^ channels open, they close very rapidly, within 10–20 ms, a process called fast or open-state inactivation. Alterations in this process are closely linked to LQT3. Figure 3A–C show the results of experiments that were designed to analyze the mutation effects on fast decay of Na^+^ current (I_Na_). In these experiments, voltage clamp pulses were applied to −20 mV to activate Na^+^ channels and representative traces are shown in Fig. 3A. A1656D mutation significantly slowed I_Na_ decay. The impact of the mutation on channel activity was more apparent in overlay of representative traces (Fig. 3A, right). These data revealed a marked increase in mutant channel activity that has failed to inactivate completely over the duration of the 500 ms test pulse. These currents are referred to as late Na^+^ channel currents (I_NaL_). The summary data show that the A1656D mutation causes a 4-fold increase in non-inactivating I_NaL_ measured at 500 ms during pulses to −20 mV (WT: I_NaL_ = 0.75%, n = 4 and A1656D: I_NaL_ = 4.11%, n = 9; p < 0.001), which is plotted as the percentage of peak current (Fig. 3B). When fast and slow components (τ1, τ2) of I_Na_ decay were assessed, both of them were significantly slowed in cells expressing A1656D mutants, compared with those of WT (Fig. 3C).

Figure 3D–F summarize the analysis on A1656D mutant on the peak current density and the voltage-dependence of inactivation and activation. A1656D mutation increased the peak Na^+^ currents at voltages ranging from −50 mV to −40 mV, while no potentiation was observed at voltages >−35 mV (Fig. 3D). This change in I–V curve was accompanied by alterations of the voltage-dependence of both the steady-state inactivation and activation (Fig. 3E,F). The steady-state inactivation was measured using the pulse protocol shown in Fig. 3E (inset). Prepulses of 500-ms duration at variable potentials (from −130 to −40 mV in 5-mV steps) preceded a 20-ms test pulse to −20 mV. The relative peak amplitudes of *I*_Na_ were plotted against prepulse voltages, and the voltages for half inactivation (V_1/2_) were calculated. The A1656D mutation evoked almost a + 20 mV depolarizing shift in the V_1/2_ of channel availability (WT: V_1/2_ = −81.9 ± 0.18 mV, n = 5 and A1656D: V_1/2_ = −68.7 ± 0.27 mV, n = 9; p < 0.01) with little or no change in the slope factor of the fitted Boltzmann relationships (WT: k = 4.5 ± 0.13 and A1656D: k = 4.1 ± 0.23). Furthermore, the mutation induced a negative shift in the steady-state activation. The activation curve was obtained by dividing the current amplitude by the electromotive force, V_m_ − E_rev_. The mean voltage for the half-maximal activation was −32.7 ± 0.68 mV (n = 5) in WT and −43 ± 0.43 mV (n = 14; p < 0.05) in A1656D mutants. We found that A1656D mutation-induced changes in Na^+^ channel activities, including an increase in I_NaL_, time dependence of gating, or voltage dependence of gating were also observed at 34 °C (Supplementary Fig. 1).

The overlap of steady-state activation and inactivation of Na^+^ channels defines arrange of voltages (i.e., window) where Na^+^ channels open, resulting in an inward Na^+^ current that could potentially depolarize the membrane potential and increase myocyte excitability^18^. Figure 3G,H show the predicted window currents of the WT and A1656D mutant Nav1.5 channels. The A1656D mutation induced an increase in the overlap of Na^+^ channel activation and inactivation by shifting the inactivation to more positive potentials and the activation to more negative potentials. The window-current voltage range is −60 to −40 mV, which corresponds to the repolarization phase. Therefore, it is possible that this small current would delay repolarization resulting in prolongation of AP. Thus, the overlap in these relationships can in part account for the LQT phenotype. Taken together, these data suggest that A1656D provoked slowed inactivation kinetics and a significant shift in the voltage dependence of gating. These alterations gave rise to an increase in the dynamic availability of the mutant channels during prolonged depolarization such as depolarization in ventricular cells during the plateau phase of the ventricular action potential (AP) underlying the QT interval of the ECG.

### Distinct pharmacological effects of A1656D mutant channels

**Figure 4:**
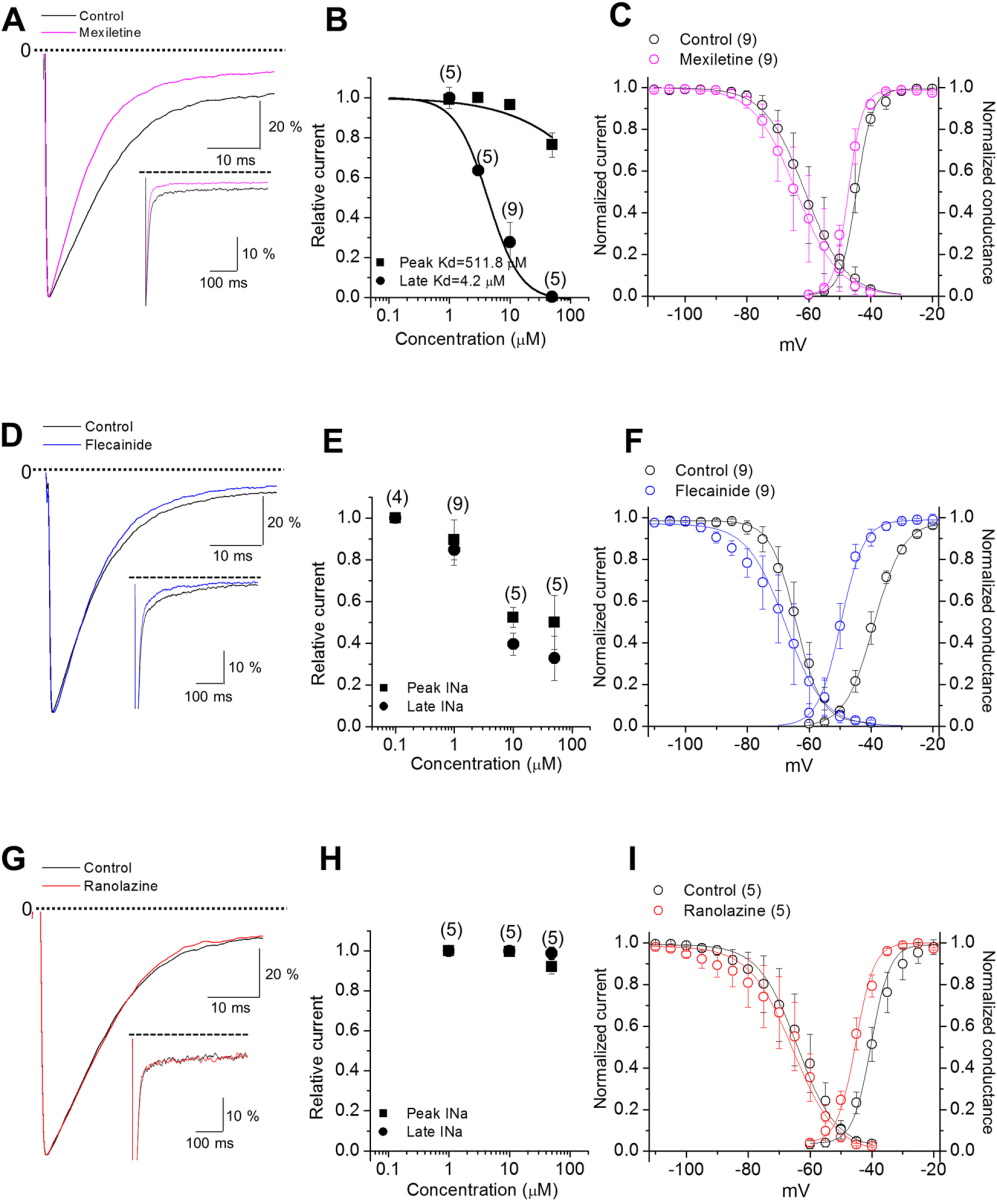
Relative sensitivities of A1656D channel current to Na^+^ channel blockers. (**A**,**D**,**G**) A1656D currents before (black in each panel) and after mexiletine (A; magenta), flecainide (**D**; blue), or ranolazine (**G**; red) were normalized to peak current and superimposed. (**B**,**E**,**H**) Data represent mean ± S.E.M. Concentration response curves of peak and late Na^+^ current during a single voltage clamp step for A1656D before and after mexiletine (**B**), flecainide (**E**), or ranolazine (**H**). (**C**,**F**,**I**) Data represent mean ± S.E.M. Steady-state inactiva(tion curves, steady-state activation curves, and window currents for A1656D before and after mexiletine (**C**), flecainide (**F**), or ranolazine (**I**).

The different responses of the patient to two Na^+^ channel blockers prompted us to investigate the effects of three different compounds, mexiletine, flecainide, and ranolazine, on A1656D mutant channel activity. Previous studies have shown that these drugs inhibited preferentially late vs. peak Na^+^ channel currents and among these, mexiletine and flecainide were effective in treating patients carrying LQT3 mutations^19,20^. The effects of mexiletine, flecainide, and ranolazine on the peak and late I_Na_ in A1656D mutant expressing cells are shown in Fig. 4. The treatment with mexiletine at a therapeutically relevant plasma concentration of 10 μM selectively inhibited late relative to the peak I_Na_ (Fig. 4A,B). However, a therapeutically relevant concentration of flecainide (1 μM, Fig. 4D,E) or ranolazine (50 μM, Fig. 4G,H) had no or little effects on late I_Na_. Further analysis showed that a therapeutically relevant concentration of mexiletine (10 μM) recovered the inactivation kinetics (Supplementary Fig. 2) although it did not affect the steady-state inactivation and steady-state activation curve of A1656D, and thus did not change the magnitude of window current (Fig. 4C). In contrast, a therapeutically relevant concentration of flecainide (1 μM) and ranolazine (50 μM) induced a left-shift in steady-state activation curve of A1656D without changes in the steady-state inactivation, leading to an overall increase in window currents (Fig. 4F,I). We also examined the effects of temperature on the pharmacological response. Consistently, late I_Na_ of A1656D mutants was effectively reduced by mexiletine but not by flecainide and ranolazine at body temperature (Supplementary Fig. 3). Further, we confirmed that mexiletine (10 μM), flecainide (1 μM), and ranolazine (50 μM) had little effects on late I_Na_ of WT Nav1.5 channels (Supplementary Fig. 4). Thus, these data suggest the selective effectiveness of mexiletine in suppressing the gain-of-function activity of A1656D.

### Rescue effects of mexiletine on A1656D mutant function in silico simulation

To gain the insight on the underlying electrophysiological characteristics of the patient, we conducted a computer simulation using human ventricular and atrial myocytes. Validation of I_Na_ model was performed by simulating the experimental voltage clamp recordings in Fig. 3. All the voltage clamp conditions including the interpulse duration were exactly same as those in experimental recordings. As shown in Supplementary Fig. 5, the time courses of fast activation and inactivation induced by depolarizing steps in simulation were nearly indistinguishable to those of experimentally obtained WT *I*_Na_ in Fig. 3A. As changes in gating are reflected in simulation to reproduce the effects of the A1656D on *I*_Na_, the time course of inactivation became slower, which were nearly equivalent to those of experimentally obtained A1656D *I*_Na_. The effects of mexiletine, flecainide, or ranolazine on the A1656D channel activity were validated by reproducing the experimental data of Fig. 4 on A1656D current traces and current-voltage (I–V) relationships during voltage clamp commands (Supplementary Figs 5 and 6).

**Figure 5:**
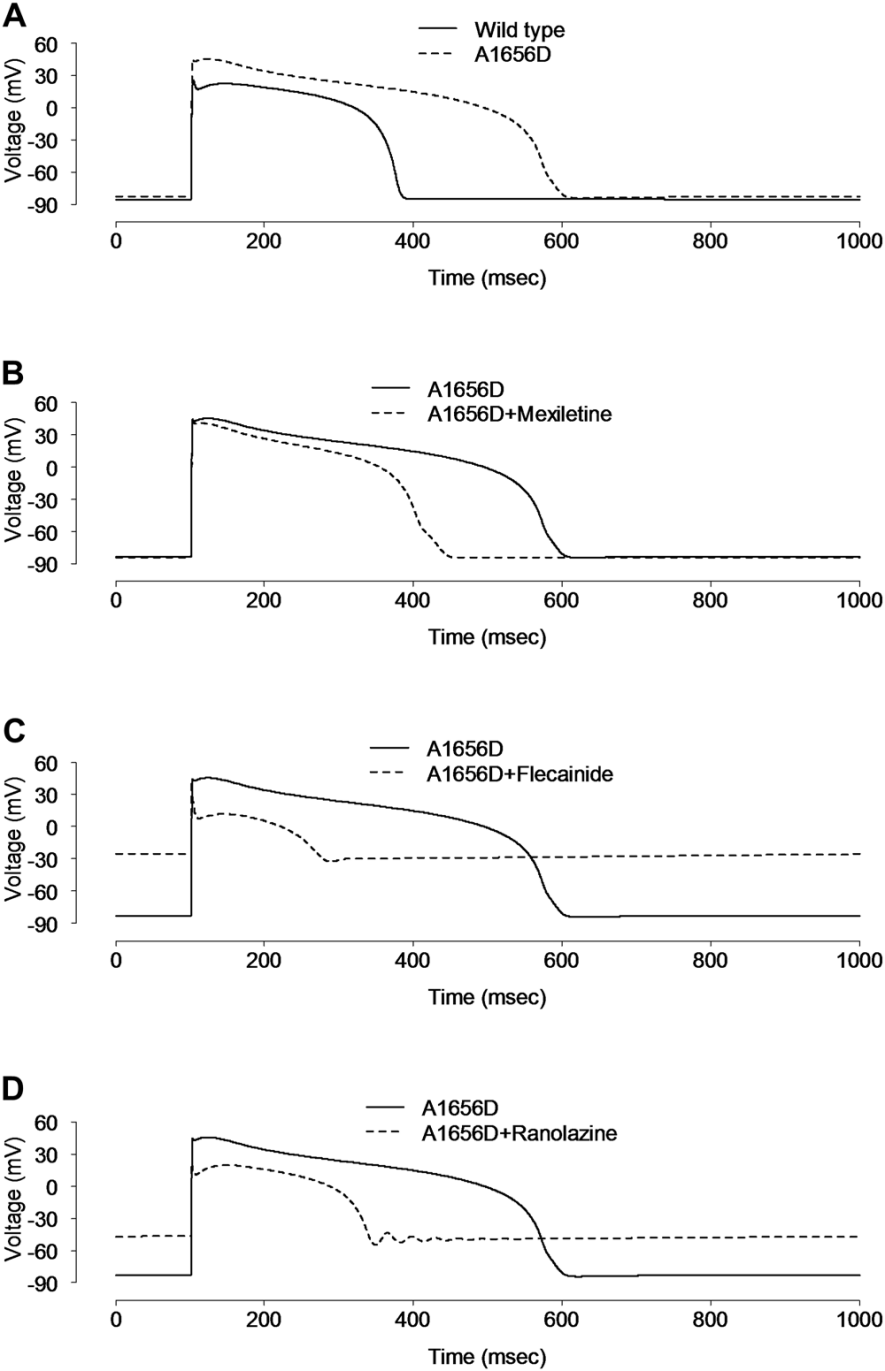
Simulation of the effect of drugs on human ventricular action potential. **(A)** The virtual AP from virtual human ventricular myocytes harboring WT SCN5A was compared with that from A1656D mutant harboring cells at 1 Hz. AP was significantly elongated in the A1656D mutant after 60 sec of stimulation at 1 Hz. B-D. Simulated effects of 10 μM mexiletine (**B**), 1 μM flecainide (**C**), and 50 μM ranolazine (**D**) on the AP of virtual human ventricular myocytes with A1656D mutation.

AP of human ventricular myocytes was simulated by using ten Tusscher’s model^21^. The resting potential was about −85.52 mV, overshoot was at +25.92 mV, and APD90 was 280.4 ms, respectively. Computer simulations of a ventricular myocytes harboring A1656D Nav1.5 showed that A1656D mutant causes a prolonged depolarization (APD90: 487.9 ms) and an increase of overshoot to +45.48 mV while the resting membrane potential was not significantly altered (Fig. 5A, −84.17 vs WT; −85.52 mV). In correlation with our results, PolyPhen-2 (http://genetics.bwh.harvard.edu/pph2/) and SIFT (https://sift.bii.a-star.edu.sg/), the functional effect prediction programs of human SNPs, predicted A1656D mutation to be damaging and deleterious, respectively (Supplementary data). Figure 5B–D, illustrates the silico simulation results of the effects with the treatment of mexiletine (10 μM), flecainide (1 μM), or ranolazine (50 μM) on myocytes expressing the mutant channel by superimposing computed APs in the absence and presence of drugs (using drug block profiles in Fig. 4) for cells paced at 1 Hz. Mexiletine treatment led to a partial recovery of APD90 from 487.9 ms to 330.5 ms at 1 Hz in A1656D ventricular myocytes, compared to the computed APD90 (=280.4 ms) of WT channel expressing cells. At 0.5 Hz stimulation frequency, the effects of A1656D mutation on APD and the response of A1656D mutants to mexiletine were similar to those at 1 Hz (Supplementary Fig. 7). When stimulation frequency was increased to 2 Hz in A1656D ventricular myocytes, APD90 was further increased with early afterdepolarizations formation, but mexiletine effectively reduced it (Supplementary Fig. 7B). We further confirmed that the reduction of APD by mexiletine was dose-dependent (Supplementary Fig. 8). This is consistent with the phenotype observed in the patient showing suppression of VT by mexiletine treatment. In contrast, flecainide and ranolazine failed to stabilize the membrane potential but rather induced the huge depolarization in A1656D ventricular myocytes in silico simulation. This disturbance might be due to enhanced I_Na_ during early repolarization of virtual ventricular myocyte model in response to these drugs (Supplementary Fig. 9).

**Figure 6:**
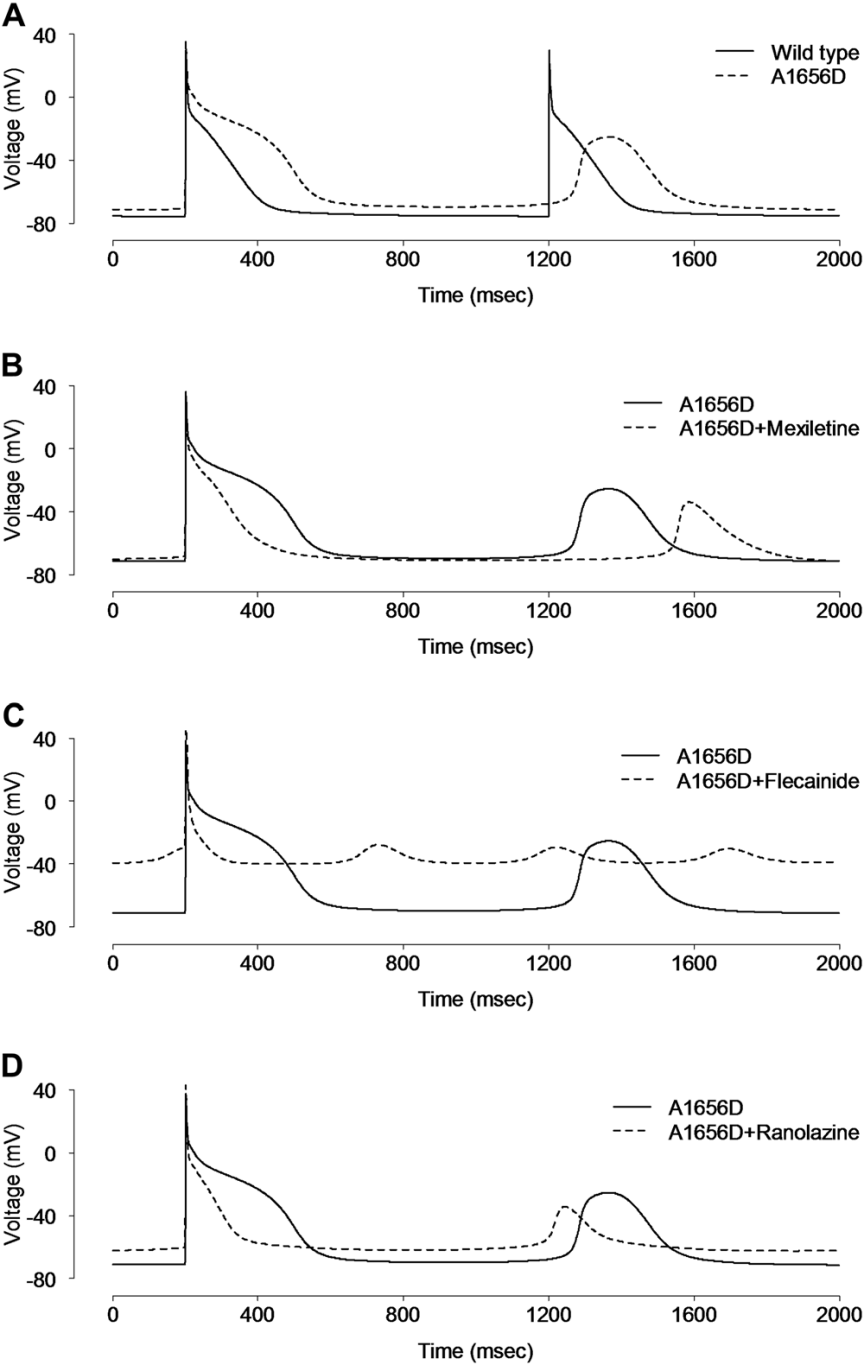
Simulation of the effect of drugs on human atrial action potential. (**A**) The virtual AP from virtual human atrial myocytes harboring WT SCN5A was compared with that from A1656D mutant harboring cells after 60 sec of stimulation at 1 Hz. The virtual human atrial myocyte with A1656D mutation exhibited prolonged APs with higher plateau and delayed after-depolarizations (DADs), compared with that of WT. (**B**–**D**) The simulated effects of mexiletine (**B**), flecainide (**C**), and ranolazine (**D**) on the AP of virtual human atrial myocytes with A1656D mutation.

In order to better understand the conversion of ventricular arrhythmia to atrial arrhythmia after mexiletine administration in this patient, the effects of the A1656D mutation were simulated by using Nygren’s model of the human atrial cell^22^. As illustrated in Fig. 6, the simulated atrial APs of A1656D prolonged APD and produced delayed after-depolarizations (DADs) (Fig. 6A). For the WT model, we could not produce DADs. Although mexiletine shortened the AP of A1656D atrial myocytes to WT values, it failed to correct the DADs, implying that mexiletine effect on I_Na_ of the human atrial cell model was not sufficient to rescue the atrial arrhythmia by A1656D Nav1.5 (Fig. 6B). Similarly in human ventricular myocyte model, neither flecainide nor ranolazine was effective in stabilizing the membrane potential but rather induced the huge depolarization in A1656D atrial myocytes in silico simulation (Fig. 6C,D). This disturbance might be due to enhanced I_Na_ during early repolarization of virtual atrial myocyte model in response to these drugs (Supplementary Fig. 10). Taken together, in silico simulation well reproduced the experimental results and allowed us to further define the disease mechanisms that is otherwise impossible to tease apart due to limitation of experimental system.

## Discussion

The results of our study demonstrate that the SCN5A A1656D mutation in a newborn perturbs the Nav1.5 channel inactivation contributing to delayed repolarization in cardiac cells. In detail, the defect in the fast inactivation caused by this mutation is similar to other previously-described LQT3 mutations such as the ΔKPQ mutation^23^. In addition to that, this mutation also produces a positive shift of the steady-state inactivation curve in the voltage-range over which the steady-state inactivation and activation overlap (window current). Each of these defects alone appears to be sufficient for the delayed repolarization for other LQT3 mutations^18,23^. Thus, it is not surprising that the patient carrying the A1656D mutation presented with QT prolongation (approximately 527 ms).

So far, more than 80 *SCN5A* mutations have been identified in patients with LQT3, and nearly 50% of them have been studied heterologously^24^. Most of these mutations are missense mutations, and are found to cause sodium channel gain-of-function, by disrupting the fast inactivation and thereby causing an abnormal sustained (or persistent) non-inactivating sodium current^23^. A1656D is located in the S4-S5 loop of D4 of Na_v_1.5 channel and this region has been already shown to be important for channel regulation. Mutations causing I_sus_ are mainly clustered in the fast inactivation-mediating regions of Na_v_1.5 channel (i.e., S4 segment of D4, the D3–D4 linker, and the cytoplasmic loops between the S4 and S5 segments of D3 and D4), or in regions that stabilize the fast inactivation (e.g., the C terminus)^24–27^. The potential mechanism of the C terminus-mediated inactivation is through interaction with the D3–D4 linker to stabilize the occlusion of the pore during inactivation^28^. The critical residues of the inactivation particle (i.e., the ball) are three consecutive amino acids near the middle of the D3-D4 linker, namely IFM (isoleucine-phenylalanine-methionine). Mutation of all three of these residues to glutamine (IFM/QQQ) abolishes the fast inactivation^29^. In the hinged lid model, depolarization exposes a binding site for the D3-D4 linker, the IFM-containing segment binds to methionine residues at 1651–1652 in the S4-S5 loop of D4. This hypothesis is supported by a report that the substitution of a pair of conserved methionine residues (M1651, M1652) in the S4-S5 loop of D4 destabilize an inactivated state^30^. Interestingly, A1656D mutation is located in the close vicinity of these methionine residues. Therefore, this mutation might also cause a decrease in the affinity of the inactivation gate for its receptor contributing to the destabilization of inactivation. Given that A1656Q mutation of SCN5A had little effects on the magnitude or voltage dependence of inactivation time constants^31^, the negatively charged residue might be important for the destabilization of inactivation. However we cannot rule out other possibilities. The alanine-to aspartic acid mutation may simply alter the tertiary structure of the S4-S5 loop or the receptor property.

Several characteristics of the clinical phenotype and therapeutic responses in this report are in alignment with those previously described for LQT3 cases in infants, including both pronounced QT prolongation^32–34^ and mixed efficacy in the controlling QT prolongation and resulting arrhythmias with Na^+^ channel blockers^32–35^. For example, whereas mexiletine was effective at controlling both 2:1 atrioventricular (AV) block in a newborn with an SCN5A (P1332L) mutation^33^, neither mexiletine nor flecainide was useful in long term control of QT prolongation in a neonate with another SCN5A mutation (R1623Q)^32^. In consideration of the variable efficacy of Na^+^ channel blockers in patients with LQT3 mutations, the development of long-QT allele-specific therapeutic strategies based on a systematic characterization of mutant channels seems to be important. In our study, we demonstrate that A1656D mutation differently responds to three Na^+^ channel blockers and that the distinct effectiveness is well correlated with the specific therapeutic responsiveness of the patient. It has been demonstrated that the structurally diverse antiarrhythmic drugs including flecainide, ranolazine and mexiletine, share overlapping binding sites in the inner-pore region of Nav1.5, which is lined by the S6 transmembrane helices and P-loop turns^36,37^. However, the variable responsiveness of the Y1767C Nav1.5 mutation to mexiletine, flecainide, and ranolazine might reflect differences in the underlying mechanisms of drug binding and Na^+^ channel inhibition^38^. This notion is well correlated with the observed responsiveness of A1656D mutation to these drugs in the current study. Currently, the mechanism leading to the ineffectiveness of ranolazine and flecainide to restore the biophysical properties of A1656D is unclear. Further study will be required to elucidate the mechanism underlying the different efficacy of these drugs.

In this study, we have analyzed a novel A1656D mutation by employing the combined approach of the experimental analysis with in silico simulation using human atrial and ventricular myocyte models. This in silico-simulation well reproduced the data obtained from the biophysical experimental analysis. In addition, it also confirmed the result from the patient treatment showing the selective efficacy of mexiletine and flecainide. Thus this study supports for the value of this type of combined approach to predict the proper therapeutics with a maximum efficacy.

## Supplementary information

Supplemental figures

Supplementary data

## Data Availability

The datasets generated and/or analyzed during the current study are available from the corresponding author on reasonable request.
