# Supplementary material for: Characterization of a novel LQT3 variant with a selective efficacy of mexiletine treatment
Source: Sci Rep. 2019 Sep 10;9:12997. doi: 10.1038/s41598-019-49450-0 (PMC6736863; doi:10.1038/s41598-019-49450-0)

Characterization of a novel LQT3 variant with a selective efficacy of mexiletine treatment

Kim: Functional study of a novel LQT3 mutation

Hyun-Ji Kim[1,3*], Bok-Geon Kim[2,3*], Jong Eun Park[4], Chang-Seok Ki[5], June Huh[6], Jae Boum Youm[7], Jong-Sun Kang[2,3], Hana Cho[1,3]

[1]Department of Physiology, [2]Department of Molecular Cell Biology, [3]Single Cell Network Research Center, Sungkyunkwan University, Suwon, Korea; [4]Department of Laboratory Medicine, Hanyang University Guri Hospital, Hanynag University College of Medicine, Guri, Republic of Korea; [5]GC Genome, Yongin, Republic of Korea; [6]Division of Pediatric Cardiology, Department of Pediatrics, Samsung Medical Center, Sungkyunkwan University School of Medicine, Seoul, Korea; [7]Department of Physiology, College of Medicine, Cardiovascular and Metabolic Disease Center, Inje University, Busan, Korea.

* These authors contributed equally to this work.

Corresponding author address:

Prof. Hana Cho MD, PhD

Department of Physiology, Sungkyunkwan University School of Medicine, 2066, Seobu-Ro, Jangan-gu, Suwon, Gyunggi-do, Korea 16419

Phone: +82-31-299-6104,

E-mail address: hanacho@skku.edu

Total number of supplementary materials: 8

Supplementary figure legends: 1

Supplementary figure: 10

Supplementary Figure Legends

Supplementary Figure 1. Comparison of channel activity of WT and A1656D mutants at RT or 34℃ . A. Whole-cell Na^+ channel currents was measured from HEK293T cells expressing WT or A1656D Nav1.5 with the β1 subunits at -20 mV (500 ms pulses applied from -120 mV holding potential at 0.2 Hz) at room temperature (left) or 34℃ (right) . WT and A1656D currents normalized to peak current and superimposed at high (right) and low (inset) gain. **B.** Bar graph representing mean±S.E.M. percentage of current remaining at 500ms with respect to peak current for both WT and A1656D Nav1.5 at room temperature (left) or 34℃ (right) . **C.** The current-voltage (**I**-V) relationship for WT and A1656D currents at room temperature (left) or 34℃ (right). **D.** Steady-state inactivation curves and steady-state activation curves were measured with WT and A1656D at room temperature (left) or 34℃ (right). The shapes of voltage step pulses are included as a figure inset. * p<0.05, ** p<0.01, Student's t-test.

Supplementary Figure 2. The effects of mexiletine (10 μM) on inactivation time constants of A1656D SCN5A in HEK293T cells. Data represent mean ± S.E.M. **P < 0.01, ***P < 0.001, Paired Student's t-test. The fast and slow time constants (Tau1, Tau2) for inactivation are plotted for A1656D Na^+ channel before and after mexiletine treatment.

Supplementary Figure 3. Relative sensitivities of A1656D Nav1.5 channel current to Na^+ channel blockers at 34℃. A,B,C. A1656D Nav1.5 currents before (black in each panel) and after 10 μM mexiletine (A; magenta), 1 μM flecainide (B; blue), or 50 μM ranolazine (C; red). I_{Na} was elicited by a 500 ms step depolarization from -120 mV to -20 mV. **D.** The proportion of I_{Na} remaining at 500 ms after treatment with mexiletine, flecainide, or ranolazine was normalized to I_{Na} in control solutions. Bar graph representing mean±S.E.M. * p<0.05, *** p<0.001, Student's t-test.

Supplementary Figure 4. Relative sensitivities of late I_{Na} of WT Nav1.5 channel to Na^+ channel blockers. A,B,C. WT Nav1.5 currents before (black in each panel) and after 10 μM mexiletine (A; magenta), 1 μM flecainide (B; blue), or 50 μM ranolazine (C; red). I_{Na} was elicited by a 500 ms step depolarization from -120 mV to -20 mV. **D.** The proportion of I_{Na} remaining at 500 ms after treatment with mexiletine, flecainide, or ranolazine was normalized to I_{Na} in control solutions. Bar graph representing mean±S.E.M. *NS*, non significant.

Supplementary Figure 5. Representative current traces of WT and A1656D Nav1.5 channel in a simulation. A. I_{Na} of WT (left) is compared with that of A1656D Nav1.5 channel (right). **B-D.** Simulated effects of mexiletine (B), flecainide (C), and ranolazine (D) on I_{Na} of A1656D mutants. The same pulse protocol in Figure 3F is employed to simulate depolarization-activated membrane currents carried by A1656D channel.

Supplementary Figure 6. Comparison of experimental and simulated I-V relationships of WT and A1656D mutants. . IV curves are compared between simulation and cell experiment. They are similar in the voltage-dependence and relative conductance. The A1656D mutation shifts the voltage-dependence to the left and increases the current amplitude in both simulation and cell experiment. Mexiletine reduces the current amplitude and shifts the voltage-dependence further to the left while flecainide and ranolazine have minimal effects on the voltage-dependence and current amplitude.

Supplementary Figure 7. Effects of stimulation frequency on action potentials in virtual human ventricular myocytes with WT SCN5A or A1656D mutant. APD50 (A) and APD90 (B) calculated at different stimulation frequencies (0.5, 1, and 2Hz) in virtual human ventricular myocytes with WT or A1656D SCN5A. 60 s of stimulation were applied before taking action potential parameters.

Supplementary Figure 8. Mexiletine reduced APDs of human cardiac myocytes model with A1656D SCN5A in a dose-dependent manner. A. Simulation of the effects of mexiletine on APs of human ventricular myocytes. **B.** Simulation of the effects of mexiletine on APs of human atrial myocytes.

Supplementary Figure 9 . Flecainide and ranolazine enhance I_{Na} during repolarization of human ventricular myocytes in a simulation. Upper panels in A and B represent membrane potential while lower panels represent I_{Na}.

Supplementary Figure 10. Flecainide and ranolazine enhance I_{Na} during repolarization of human atrial myocytes in a simulation. Upper panels in A and B represent membrane potential while lower panels represent I_{Na}.

Supplementary Figure 1

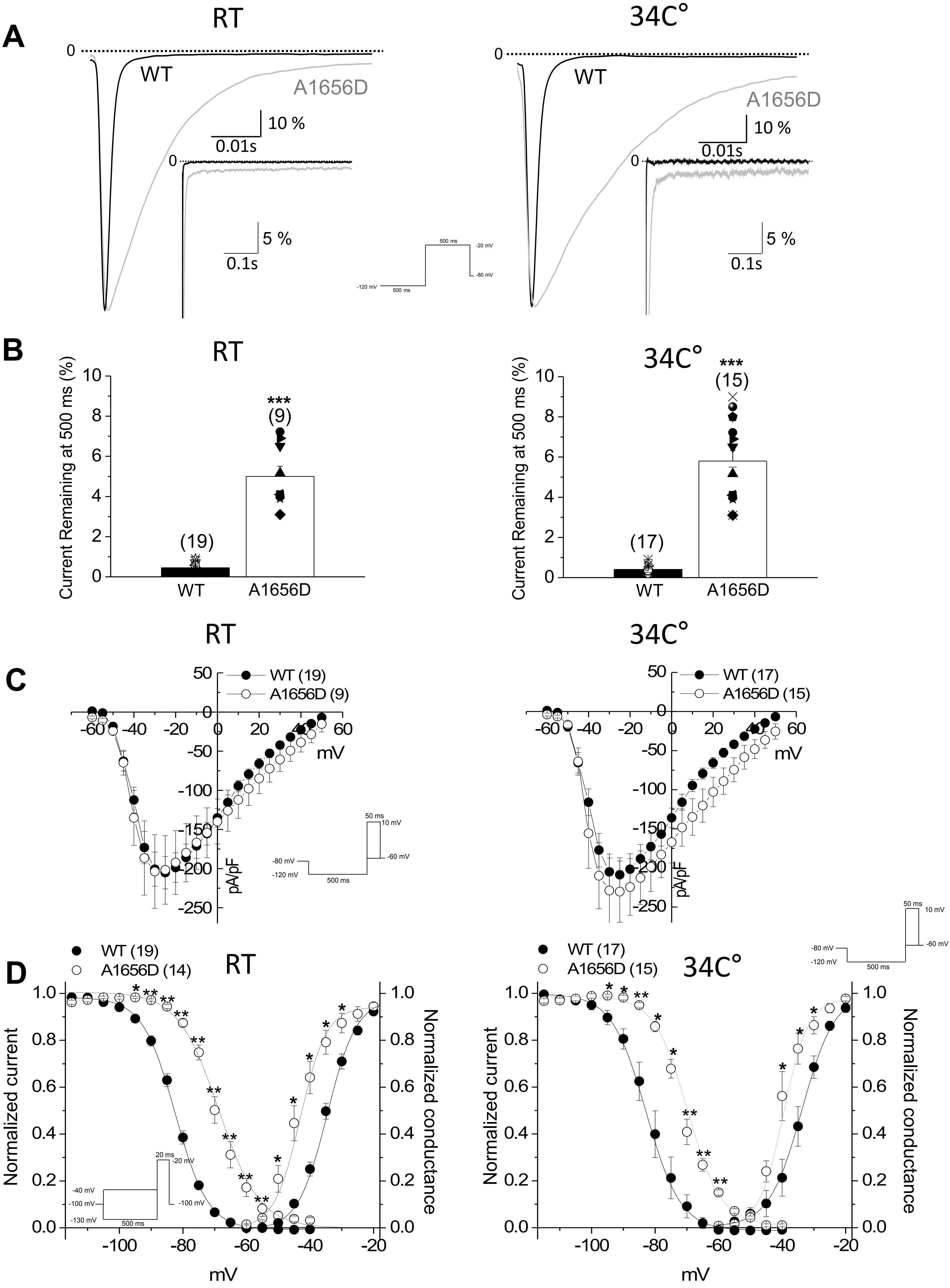

Supplementary Figure 2

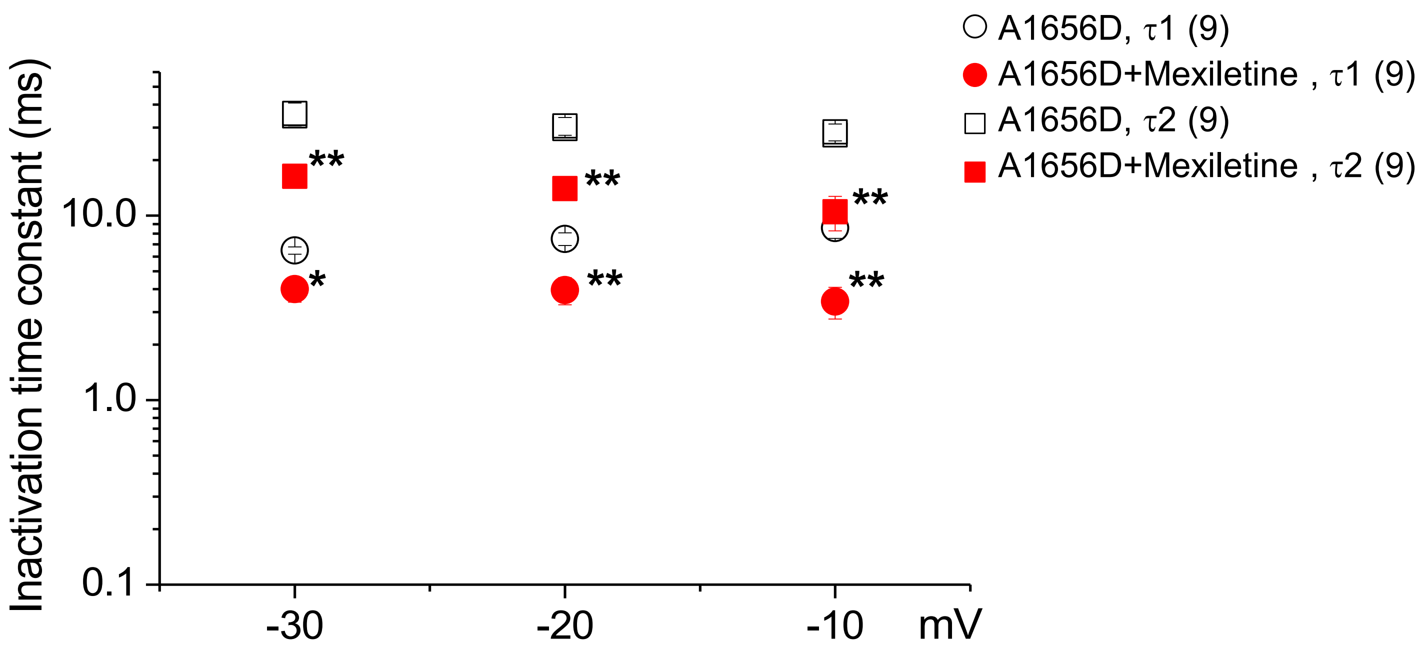

Supplementary Figure 3

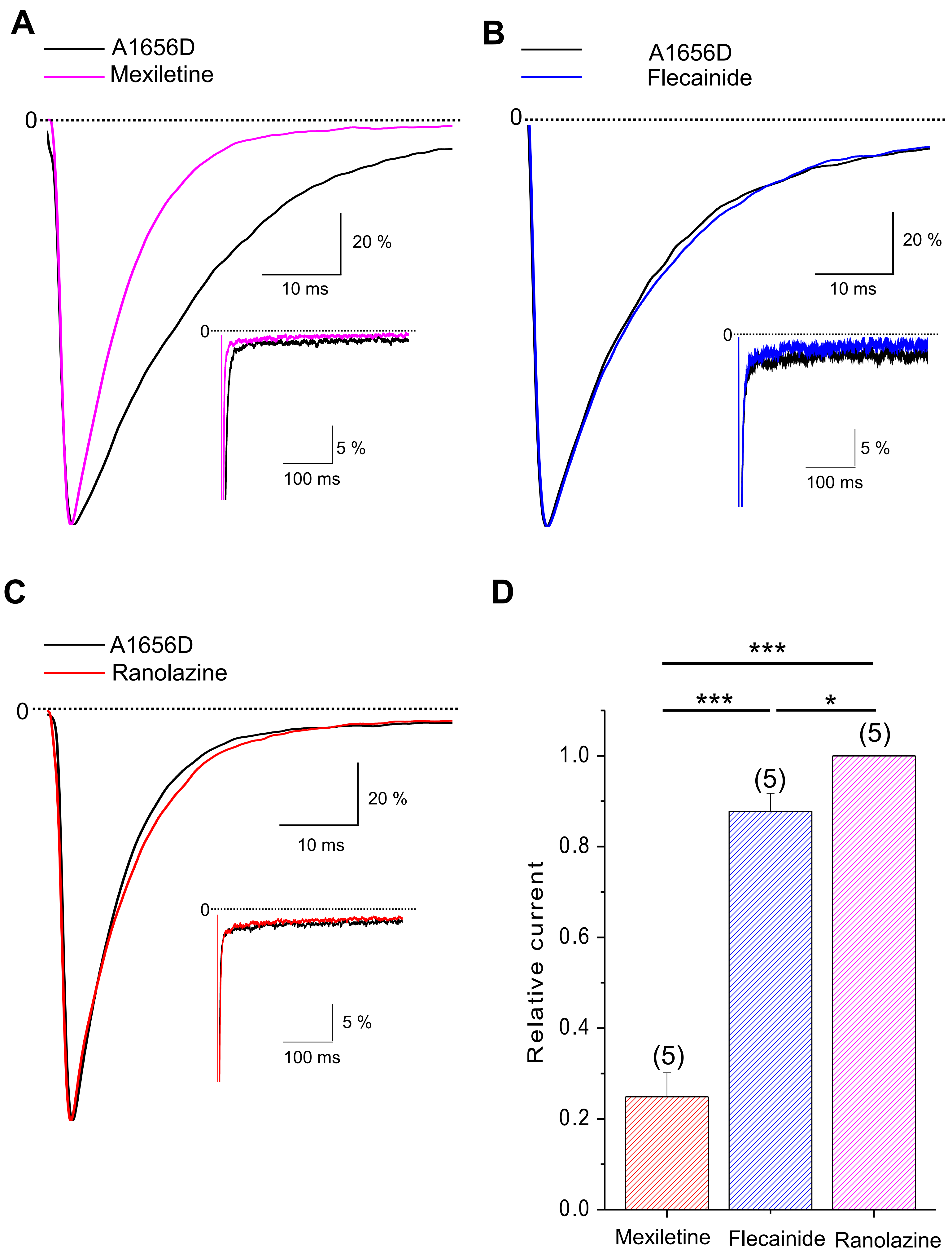

Supplementary Figure 4

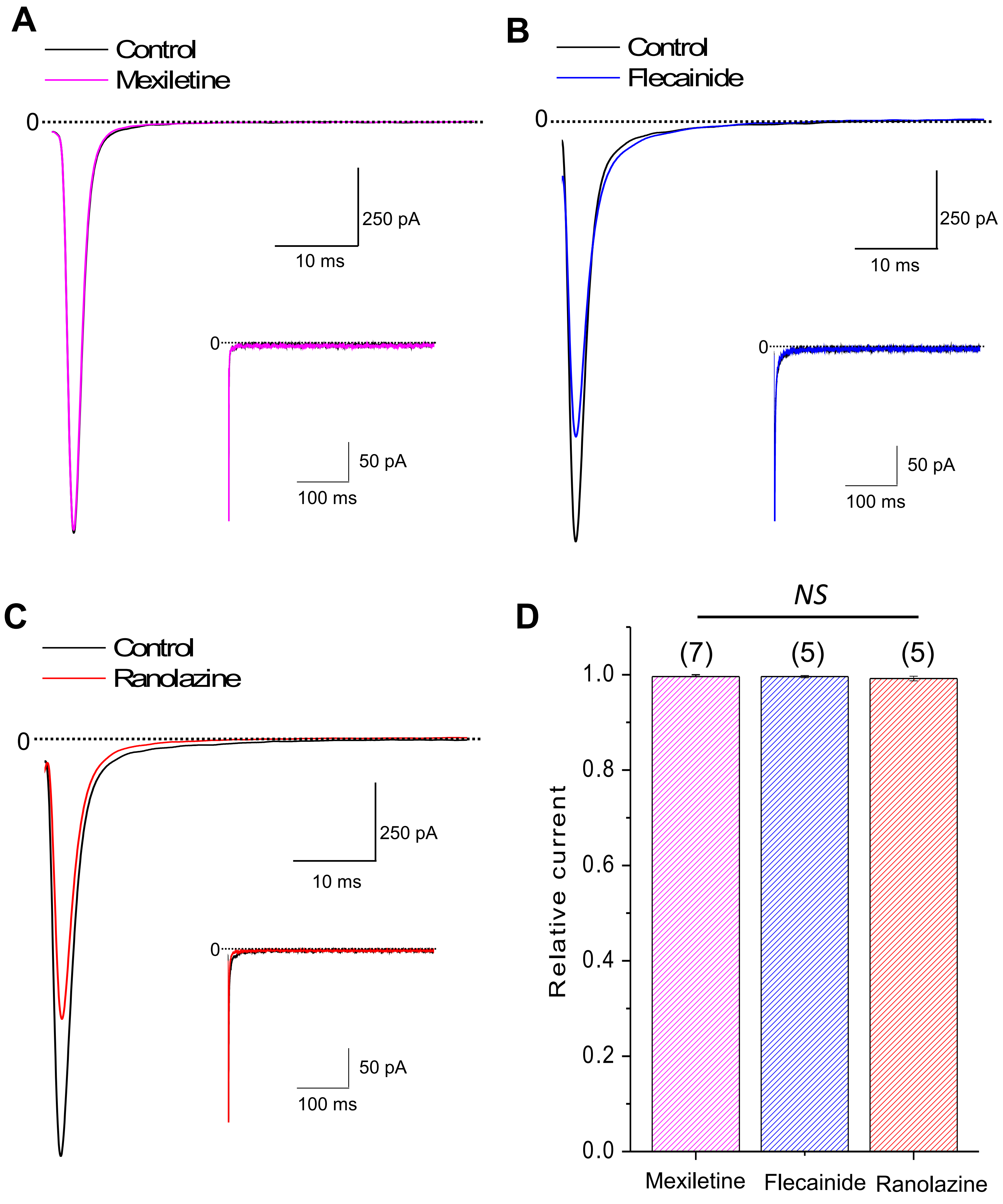

Supplementary Figure 5

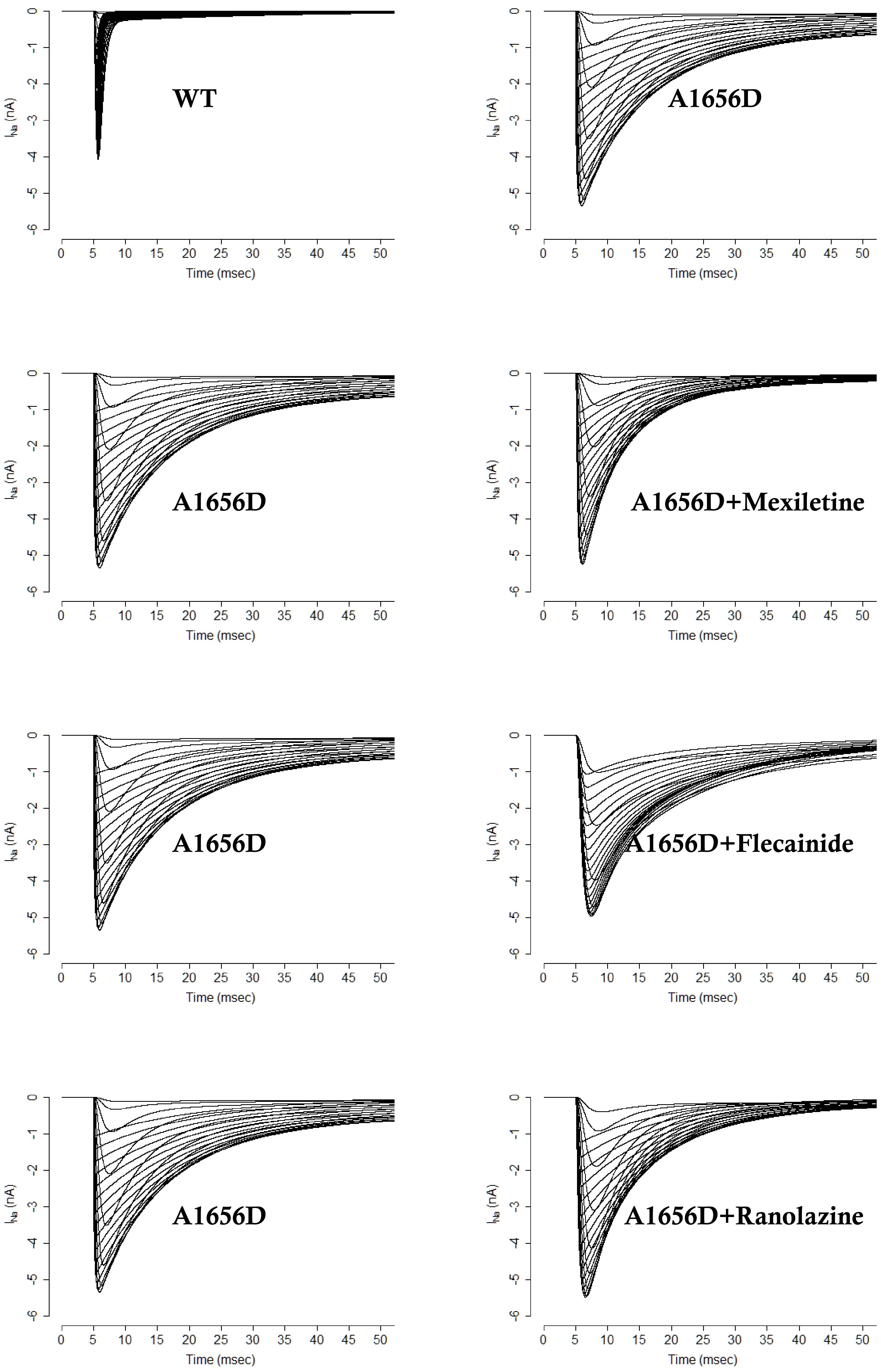

Supplementary Figure 6

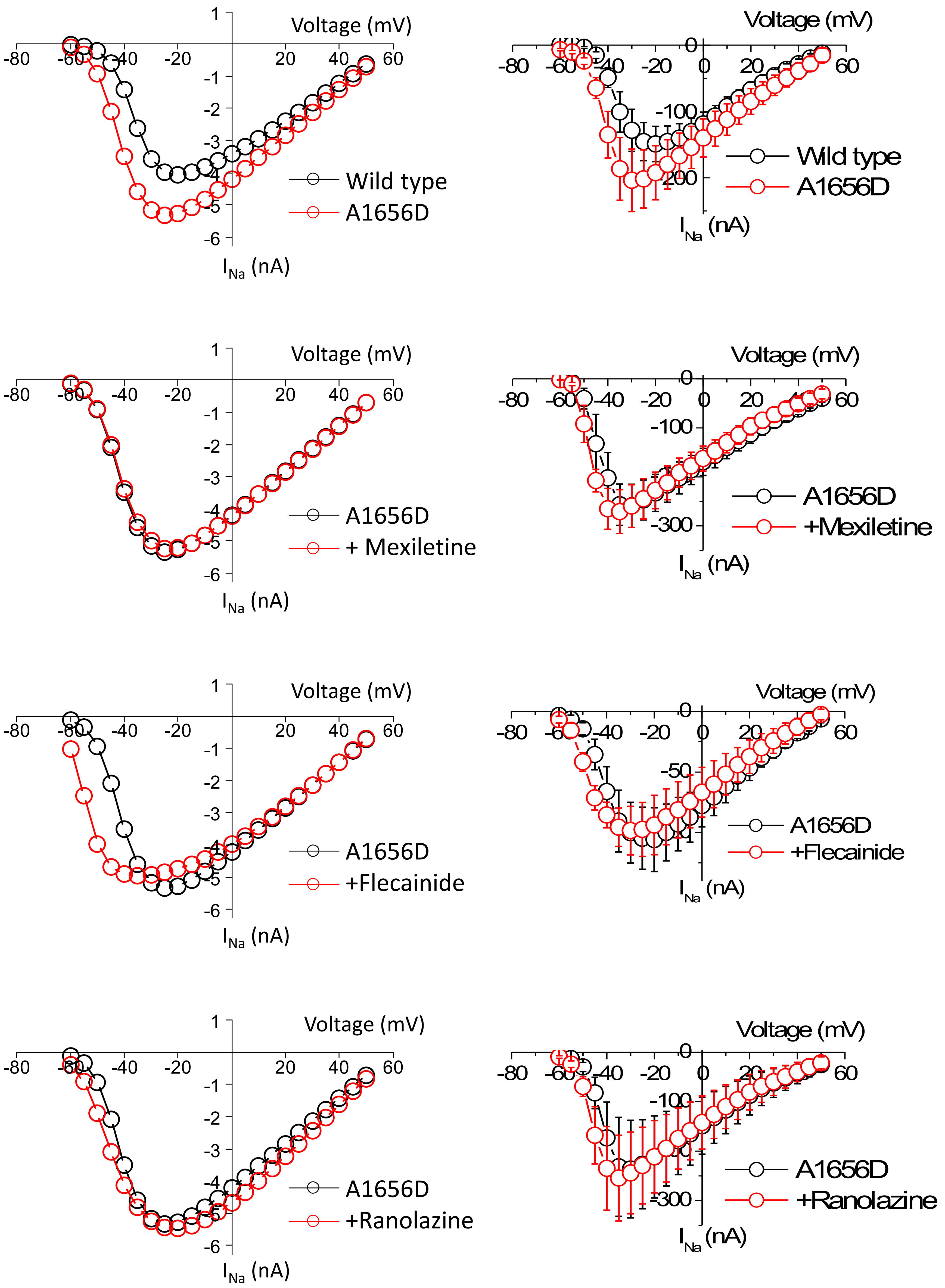

Supplementary Figure 7

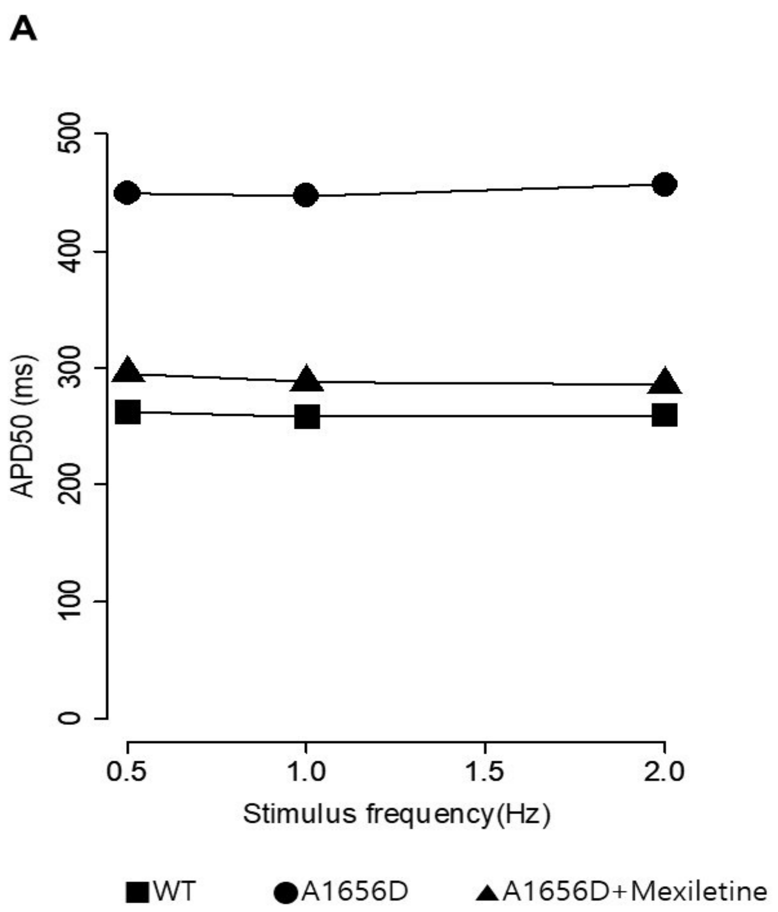

Supplementary Figure 8

Ventricular myocyte model

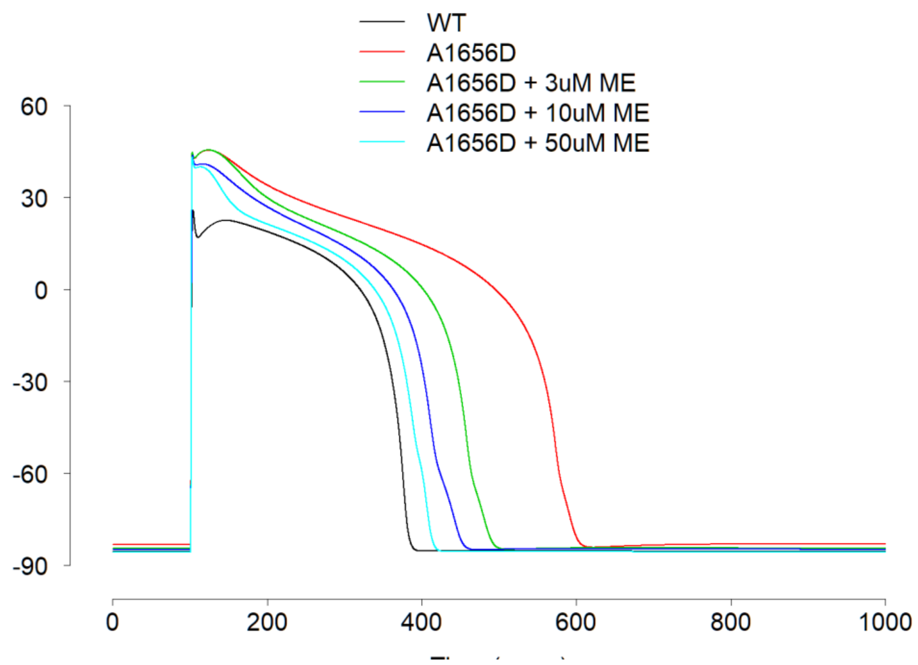

Atrial myocyte model

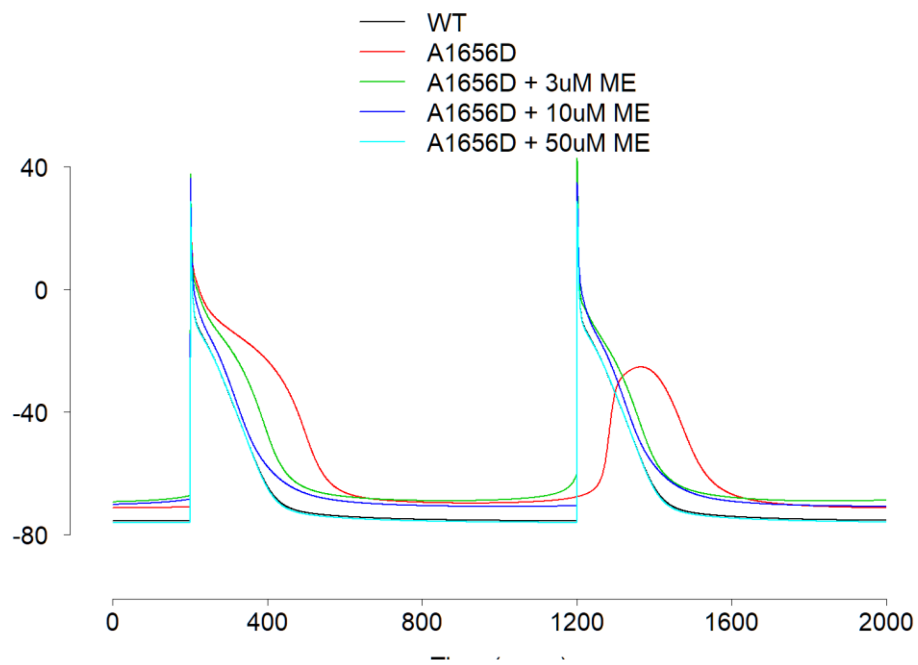

Supplementary Figure 9

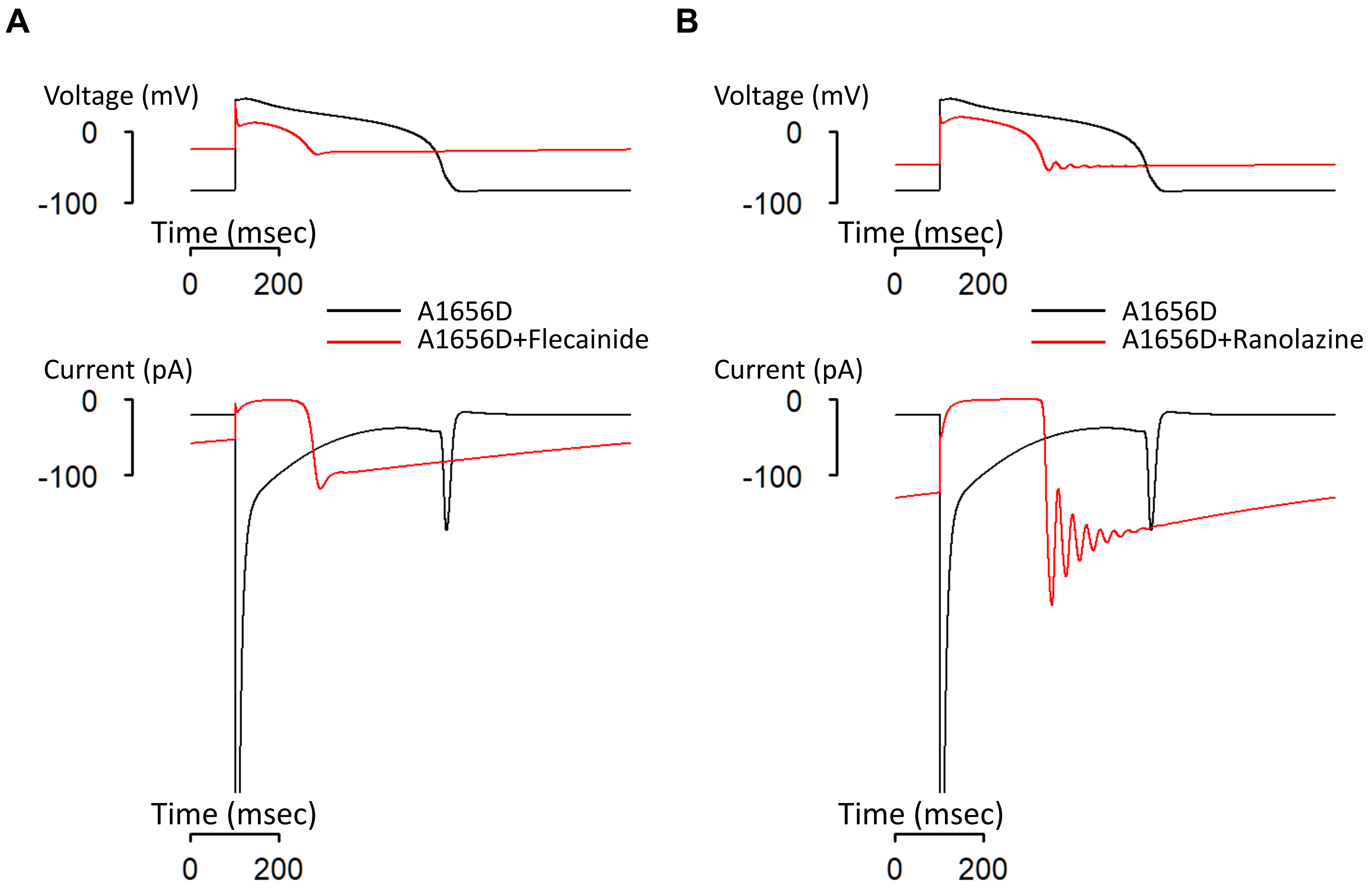

Supplementary Figure 10

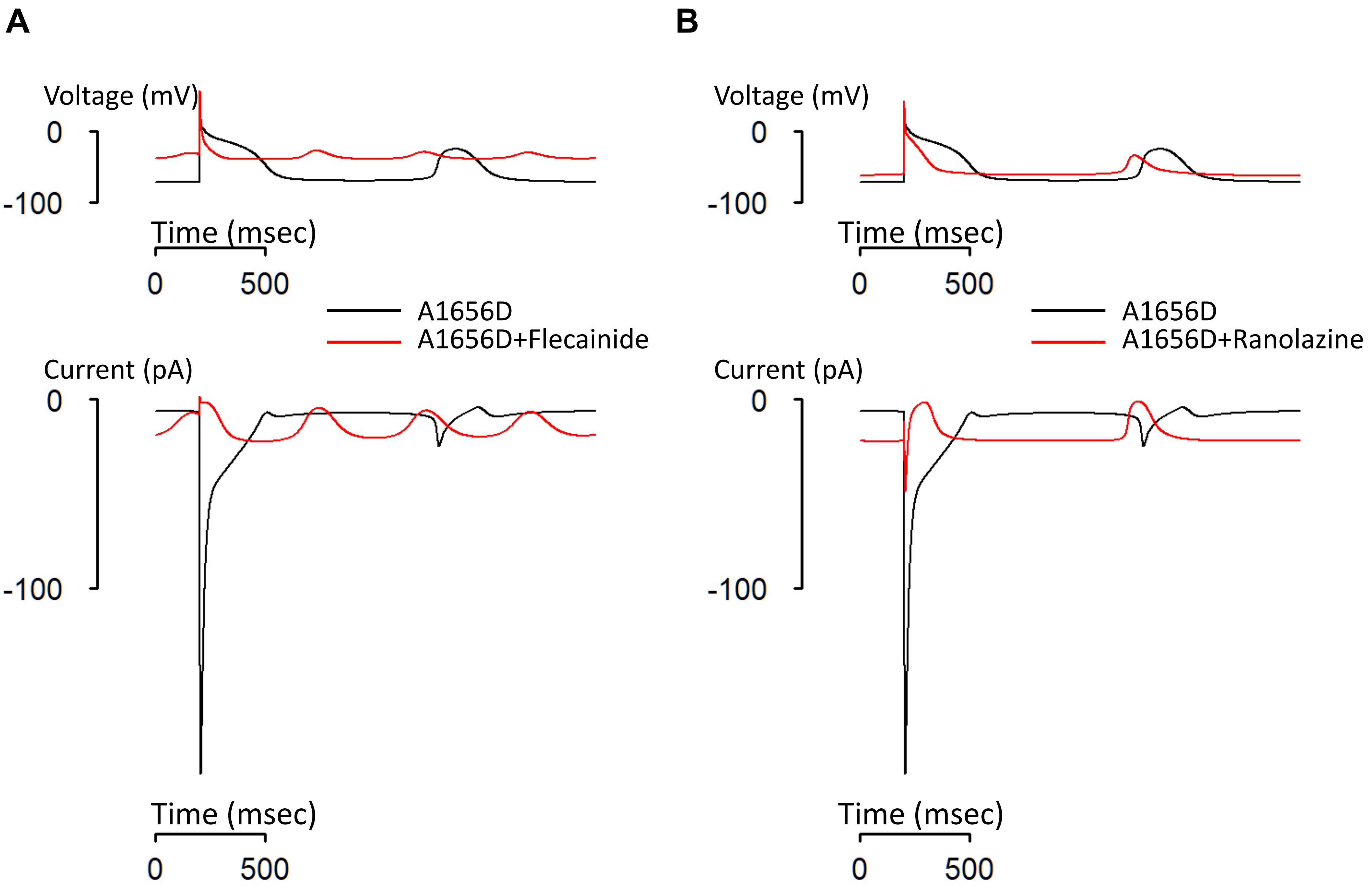

Supplement: Supplementary file 1 — Supplemental figures [file 41598_2019_49450_MOESM1_ESM.pdf]
