# Supplementary material for: Characterization of a novel LQT3 variant with a selective efficacy of mexiletine treatment
Source: Sci Rep. 2019 Sep 10;9:12997. doi: 10.1038/s41598-019-49450-0 (PMC6736863; doi:10.1038/s41598-019-49450-0)
Supplement: Supplementary file 2 — Supplementary data [file 41598_2019_49450_MOESM2_ESM.pdf]

Characterization of a novel LQT3 variant with a selective efficacy of mexiletine treatment

Kim: Functional study of a novel LQT3 mutation

Hyun-Ji Kim[1,3*], Bok-Geon Kim[2,3*], Jong Eun Park[4], Chang-Seok Ki[5], June Huh[6], Jae Boum Youm[7], Jong-Sun Kang[2,3], Hana Cho[1,3]

[1]Department of Physiology, [2]Department of Molecular Cell Biology, [3]Single Cell Network Research Center, Sungkyunkwan University, Suwon, Korea; [4]Department of Laboratory Medicine, Hanyang University Guri Hospital, Hanynag University College of Medicine, Guri, Republic of Korea; [5]GC Genome, Yongin, Republic of Korea; [6]Division of Pediatric Cardiology, Department of Pediatrics, Samsung Medical Center, Sungkyunkwan University School of Medicine, Seoul, Korea; [7]Department of Physiology, College of Medicine, Cardiovascular and Metabolic Disease Center, Inje University, Busan, Korea.

* These authors contributed equally to this work.

Corresponding author address:

Prof. Hana Cho MD, PhD

Department of Physiology, Sungkyunkwan University School of Medicine, 2066, Seobu-Ro, Jangan-gu, Suwon, Gyunggi-do, Korea 16419

Phone: +82-31-299-6104,

E-mail address: hanacho@skku.edu

1. PolyPhen-2

Protein sequence in FASTA format:

>NP_932173.1 sodium channel protein type 5 subunit alpha isoform a
[Homo sapiens]

MANFLLPRGTSSFRRFTRESLAAIEKRMAEKQARGSTTLQESREGLPEEEAPRPQL
DLQASKKLPDLYGNPPQELIGEPLEDLDPFYSTQKTFIVLNKGKTIFRFSATNALYVL
SPFHPIRRAAVKILVHSLFNMLIMCTILTNCVFMAQHDPPPWTKYVEYTFTAIYTFESL
VKILARGFCLHAFTFLRDPWNWLDFSVIIMAYTTEFVDLGNVSALRTFRVLRALKTIS
VISGLKTIVGALIQSVKKLADVMVLTVFCLSVFALIGLQLFMGNLRHKCVRNFTALNG
TNGSVEADGLVWESLDLYLSDPENYLLKNGTSDVLLCGNSSDAGTCPEGYRCLKA
GENPDHGYTSFDSFAWAFLALFRLMTQDCWERLYQQTLRSAGKIYMIFFMLVIFLG
SFYLVNLILAVVAMAYEEQNQATIAETEEKEKRFQEAMEMLKKEHEALTIRGVDTVS
RSSLEMSPLAPVNSHERRSKRRKRMSSGTEECGEDRLPKSDSEDGPRAMNHLSL
TRGLSRTSMKPRSSRGSIFTFRRRDLGSEADFADDENSTAGESESHHTSLLVPWPL
RRTSAQGQPSPGTSAPGHALHGKKNSTVDCNGVVSLLGAGDPEATSPGSHLLRP
VMLEHPPDTTTPSEEPGGPQMLTSQAPCVDGFEEPGARQRALSAVSVLTSALEEL
EESRHKCPPCWNRLAQRYLIWECCPLWMSIKQGVKLVVMDPFTDLTITMCIVLNTLF
MALEHYNMTSEFEEMLQVGNLVFTGIFTAEMTFKIIALDPYYYFQQGWNIFDSIIVILS
LMELGLSRMSNLSVLRSFRLLRVFKLAKSWPTLNTLIKIIGNSVGALGNLTLVLAIIVFI
FAVVGMQLFGKNYSELRDSDSGLLPRWHMMDFFHAFLIIFRILCGEWIETMWDCME
VSGQSLCLLVFLLVMVIGNLVVLNLFLALLLSSFSADNLTAPDEDREMNNLQLALARI
QRGLRFVKRTTWDFCCGLLRQRPQKPAALAAQGQLPSCIATPYSPPPPETEKVPP
TRKETRFEEGEQPGQGTPGDPEPVCVPIAVAESDTDDQEEDEENSLGTEEESSKQ
QESQPVSGGPEAPPDSRTWSQVSATASSEAEASASQADWRQQWKAEPQAPGCG
ETPEDSCSEGSTADMTNTAELLEQIPDLGQDVKDPEDCFTEGCVRRCPCCAVDTT
QAPGKVWWRLRKTCYHIVEHSWFETFIIFMILLSSGALAFEDIYLEERKTIKVLLEYA
DKMFTYVFVLEMLLKWVAYGFKKYFTNAWCWLDFLIVDVSLVSLVANTLGFAEMGPI
KSLRTLRALRPLRALSRFEGMRVVVNALVGAIPSIMNVLLVCLIFWLIFSIMGVNLFAG
KFGRCINQTEGDLPLNYTIVNNKSQCESLNLTGELYWTKVKVNFDNVGAGYLALLQ
VATFKGWMDIMYAAVDSRGYEEQPQWEYNLYMYIYFVIFIIFGSFFTLNLFIGVIIDNF
NQQKKKLGGQDIFMTEEQKKYYNAMKKLGSKKPQKPIPRPLNKYQGFIFDIVTKQA
FDVTIMFLICLNMVTMMVETDDQSPEKINILAKINLLFVAIFTGECIVKLAALRHYYFTN
SWNIFDFVVVILSIVGTVLSDIIQKYFFSPTLFRVIRLARIGRILRLIRGAKGIRTLLFALM
MSLPALFNIGLLLFLVMFIYSIFGMANFAYVKWEAGIDDMFNFQTFANSMLCLFQITTS
AGWDGLLSPILNTGPPYCDPTLPNSNGSRGDCGSPAVGILFFTTYIIISFLIVVNMYIAI
ILENFSVATEESTEPLSEDDFDMFYEIWEKFDPEATQFIEYSVLSDFADALSEPLRIAK
PNQISLINMDLPMVSGDRIHCMDILFAFTKRVLGESGEMDALKIQMEEKFMAANPSK
ISYEPITTTLRRKHEEVSAMVIQRAFRRHLLQRSLKHASFLFRQQAGSGLSEEDAPE
REGLIAYVMSENFSRPLGPPSSSSISSTSFPPSYDSVTRATSDNLQVRGSDYSHSE
DLADFPPSPDRDRESIV

Position: 1656

Substitution: AA1-A; AA2-D

Results:

Prediction Model	Score		Prediction Report
	Sensitivity	Specificity	
HumDiv	1.000		**Probably Damaging**
	0.00	1.00	
HumVar	0.999		**Probably Damaging**
	0.09	0.99	

2. SIFT

Protein query sequence:

>NP_932173.1 sodium channel protein type 5 subunit alpha isoform a
[Homo sapiens]

MANFLLPRGTSSFRRFTRESLAAIEKRMAEKQARGSTTLQESREGLPEEEAPRPQL
DLQASKKLPDLYGNPPQELIGEPLEDLDPFYSTQKTFIVLNKGKTIFRFSATNALYVL
SPFHPIRRAAVKILVHSLFNMLIMCTILTNCVFMAQHDPPPWTKYVEYTFTAIYTFESL
VKILARGFCLHAFTFLRDPWNWLDFSVIIMAYTTEFVDLGNVSALRTFRVLRALKTIS
VISGLKTIVGALIQSVKKLADVMVLTVFCLSVFALIGLQLFMGNLRHKCVRNFTALNG
TNGSVEADGLVWESLDLYLSDPENYLLKNGTSDVLLCGNSSDAGTCPEGYRCLKA
GENPDHGYTSFDSFAWAFLALFRLMTQDCWERLYQQTLRSAGKIYMIFFMLVIFLG
SFYLVNLILAVVAMAYEEQNQATIAETEEKEKRFQEAMEMLKKEHEALTIRGVDTVS
RSSLEMSPLAPVNSHERRSKRRKRMSSGTEECGEDRLPKSDSEDGPRAMNHLSL
TRGLSRTSMKPRSSRGSIFTFRRRDLGSEADFADDENSTAGESESHHTSLLVPWPL
RRTSAQGQPSPGTSAPGHALHGKKNSTVDCNGVVSLLGAGDPEATSPGSHLLRP
VMLEHPPDTTTPSEEPGGPQMLTSQAPCVDGFEEPGARQRALSAVSVLTSALEEL
EESRHKCPPCWNRLAQRYLIWECCPLWMSIKQGVKLVVMDPFTDLTITMCIVLNTLF
MALEHYNMTSEFEEMLQVGNLVFTGIFTAEMTFKIIALDPYYYFQQGWNIFDSIIVILS
LMELGLSRMSNLSVLRSFRLLRVFKLAKSWPTLNTLIKIIGNSVGALGNLTLVLAIIVFI
FAVVGMQLFGKNYSELRDSDSGLLPRWHMMDFFHAFLIIFRILCGEWIETMWDCME
VSGQSLCLLVFLLVMVIGNLVVLNLFLALLLSSFSADNLTAPDEDREMNNLQLALARI
QRGLRFVKRTTWDFCCGLLRQRPQKPAALAAQGQLPSCIATPYSPPPPETEKVPP
TRKETRFEEGEQPGQGTPGDPEPVCVPIAVAESDTDDQEEDEENSLGTEEESSKQ
QESQPVSGGPEAPPDSRTWSQVSATASSEAEASASQADWRQQWKAEPQAPGCG
ETPEDSCSEGSTADMTNTAELLEQIPDLGQDVKDPEDCFTEGCVRRCPCCAVDTT
QAPGKVWWRLRKTCYHIVEHSWFETFIIFMILLSSGALAFEDIYLEERKTIKVLLEYA
DKMFTYVFVLEMLLKWVAYGFKKYFTNAWCWLDFLIVDVSLVSLVANTLGFAEMGPI
KSLRTLRALRPLRALSRFEGMRVVVNALVGAIPSIMNVLLVCLIFWLIFSIMGVNLFAG
KFGRCINQTEGDLPLNYTIVNNKSQCESLNLTGELYWTKVKVNFDNVGAGYLALLQ
VATFKGWMDIMYAAVDSRGYEEQPQWEYNLYMYIYFVIFIIFGSFFTLNLFIGVIIDNF
NQQKKKLGGQDIFMTEEQKKYYNAMKKLGSKKPQKPIPRPLNKYQGFIFDIVTKQA
FDVTIMFLICLNMVTMMVETDDQSPEKINILAKINLLFVAIFTGECIVKLAALRHYYFTN
SWNIFDFVVVILSIVGTVLSDIIQKYFFSPTLFRVIRLARIGRILRLIRGAKGIRTLLFALM
MSLPALFNIGLLLFLVMFIYSIFGMANFAYVKWEAGIDDMFNFQTFANSMLCLFQITTS
AGWDGLLSPILNTGPPYCDPTLPNSNGSRGDCGSPAVGILFFTTYIIISFLIVVNMYIAI
ILENFSVATEESTEPLSEDDFDMFYEIWEKFDPEATQFIEYSVLSDFADALSEPLRIAK
PNQISLINMDLPMVSGDRIHCMDILFAFTKRVLGESGEMDALKIQMEEKFMAANPSK
ISYEPITTTLRRKHEEVSAMVIQRAFRRHLLQRSLKHASFLFRQQAGSGLSEEDAPE
REGLIAYVMSENFSRPLGPPSSSSISSTSFPPSYDSVTRATSDNLQVRGSDYSHSE
DLADFPPSPDRDRESIV

The substitutions of interest: A1656D

Database to search: UniProt-SwissProt + TrEMBL 2010_09

Median conservation of sequences: 3.00
Remove sequences more than (90) percent identical to query

Results:
Substitution at pos 1656 from A to D is predicted to AFFECT PROTEIN FUNCTION with a score of 0.00.
Median sequence conservation: 3.04
Sequences represented at this position: 129
*Amino acids with probabilities <0.05 are predicted to be deleterious.
